# Human organoids-on-chips for biomedical research and applications

**DOI:** 10.7150/thno.90492

**Published:** 2024-01-01

**Authors:** Hui Wang, Xiufan Ning, Feng Zhao, Hui Zhao, Dong Li

**Affiliations:** 1 Department of Interventional & Vascular Surgery, Affiliated Hospital of Nantong University, Medical School of Nantong University, Nantong 226001, China.; 2 College of Basic Medical Sciences, Dalian Medical University, Dalian 116044, China.; 3 Dalian Institute of Chemical Physics, Chinese Academy of Sciences, Dalian 116023, China.

**Keywords:** organoids-on-chips, organs-on-chips, drug discovery, precision medicine, living organoid biobank

## Abstract

Human organoids-on-chips (OrgOCs) are the synergism of human organoids (HOs) technology and microfluidic organs-on-chips (OOCs). OOCs can mimic extrinsic characteristics of organs, such as environmental clues of living tissue, while HOs are more amenable to biological analysis and genetic manipulation. By spatial cooperation, OrgOCs served as 3D organotypic living models allowing them to recapitulate critical tissue-specific properties and forecast human responses and outcomes. It represents a giant leap forward from the regular 2D cell monolayers and animal models in the improved human ecological niche modeling. In recent years, OrgOCs have offered potential promises for clinical studies and advanced the preclinical-to-clinical translation in medical and industrial fields. In this review, we highlight the cutting-edge achievements in OrgOCs, introduce the key features of OrgOCs architectures, and share the revolutionary applications in basic biology, disease modeling, preclinical assay and precision medicine. Furthermore, we discuss how to combine a wide range of disciplines with OrgOCs and accelerate translational applications, as well as the challenges and opportunities of OrgOCs in biomedical research and applications.

## 1. Introduction

Humans have long wondered about how human organs form and develop within a complicated environment throughout the entire life cycle. Moreover, it remains mysterious how the human organs are affected after the onset of disease, and to what extent it can be cured by drug and cell therapy. Our current understanding largely relies on conventional cell cultures and animal models. However, these models are greatly limited due to the species divergence as well as differences in organ architecture and function. Therefore, it is highly anticipated to establish highly biomimetic human organ models for a broad range of applications including biological study, disease modeling, drug screening, personalized medicine, etc. With the development of multiple disciplines including biomaterial, chemistry, computer and mathematics, scientists have presented various reliable human organ models 1-5. As emerging technologies, OOCs and HOs have attracted worldwide attention due to their unique advantages.

OOC is an *in vitro* microphysiological system that can accurately incorporate crucial microenvironment parameters of living organs, including biochemical factors, physical factors, systematic interactions between multiple organs or organ-matrix, essentially representing organ-level 'synthetic biology'. Typically, shear stress, dynamic fluid, mechanical tension, oxygen gradient, bioscaffolds, electrical and optical signals can be easily manipulated in a spatiotemporal controllable manner 6-8. Currently, breathing lung, peristaltic intestine, rhythmic heart, folded brain, and even body axis (e.g., liver-islet axis, gut-kidney axis, and gut-microbe-brain axis) have been developed to model human physiopathologic ecosystems, including organogenesis, host-immune response, and drug-organ interplay 9-14. Differ from OOC, the concept of HOs is proposed by researchers in stem cell biology, which can recapitulate many biological parameters (e.g., multicellular paracrine and autocrine effect, tissue-specific polarization and cell-matrix interactions) and be used for parsing cell fates during organogenesis and studying the mechanisms that underlie stem cell self-assembly, proliferation, differentiation, and stemness maintenance. The bionic multicellular types and high fidelity and maturity are the highlights of HOs 15-17. Notably, gene editing, omics, artificial intelligence and biomaterials have reinforced the rising impacts of HOs in life sciences and biomedicine 18-24. These burgeoning technologies expand the utilization of HOs and offer more possibilities for tissue regeneration, disease treatment, drug discovery and personalized medicine 25-29. As a new generation of organ chips, OrgOC has just been proposed in recent years. The cutting-edge OrgOC will further promote the engineering technologies applicable in biomedicine by maximizing the potential of OOCs with higher integration and HOs with higher fidelity. The past few years have witnessed the rapid development and widespread application of OrgOCs. On an international scale, diverse OrgOCs with organ-level structures and functional units have been established, involving most parts of the body such as reproductive systems, endocrine systems, vascular systems, nervous systems and so on 30-35.

**Figure 1:**
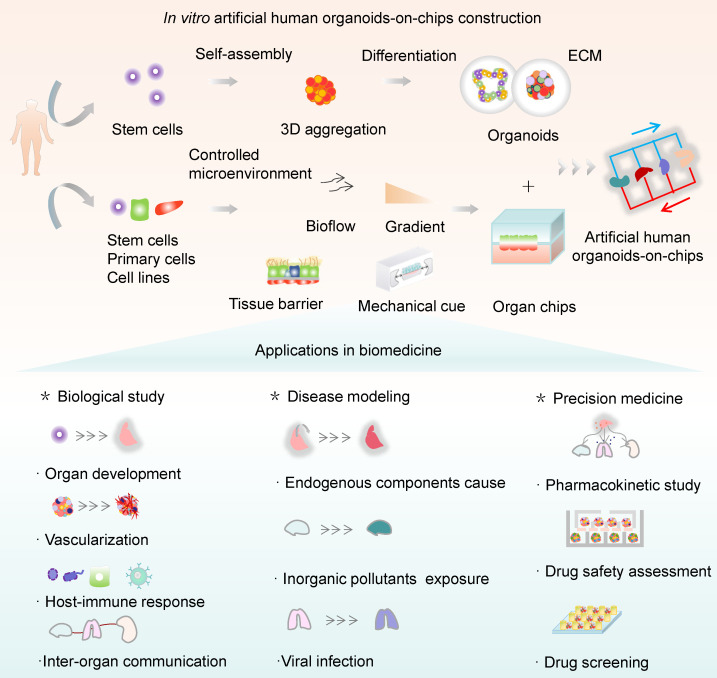
Schematic for *in vitro* human organoids-on-chips models for biomedicine. Human organoids are 3D multicellular tissue constructs derived from human stem cells (e.g., pluripotent stem cells or adult stem cells) through self-assembly, retaining certain key features of their organ counterparts. Organ chips are microengineered microfluidic cell culture setups that can recreate cell microenvironment (e.g., bioflow, gradient, tissue barrier and mechanical cue) in a controlled manner. Human organoids-on-chips (OrgOCs) are opening up a new frontier of biomedical research by integrating organoids and organ chips technology. These physiologically relevant microsystems can be widely used in the biological study (e.g., organ development, vascularization, host-immune response and inter-organ communication), disease modeling (e.g., endogenous components cause, inorganic pollutants exposure and viral infection) and precision medicine (e.g., pharmacokinetic study, drug safety assessment and drug screening).

This systemic review aims to highlight the recent progress of the OrgOC technology and its widespread applications in basic research and translational medicine (Figure 1). First, a snapshot of the remarkable hallmarks of OOCs, HOs, and OrgOCs that determine the physiological relevance of *in vitro* human organ models was given from different perspectives. Second, the biomedical applications of OrgOCs in biological study, tissue regeneration, disease modeling, disease therapy, precision medicine, and drug development were introduced in detail. Third, we discuss the current limitations and future perspectives of OrgOCs in biomedical applications. We believe that the integration of multidisciplinary technologies (e.g., biomaterial, 3D printing, gene editing, artificial intelligence, and multi-omics analysis) will greatly enhance the potential of OrgOCs in biomedical research and applications.

## 2. Snapshot of OOCs, HOs, and OrgOCs

Living organisms are regulated by the dynamic crosstalk of the inherent genetic reprograms of cells and the external cues. These elements can be spatiotemporally controlled and play a pivotal role in the construction of OrgOCs. OrgOCs have integrated OOC and HO technologies to build human 3D organ models with more representative tissue-specific properties and critical functions at an organ level. OOC and HO technologies represent two fundamentally different but complementary approaches toward a common goal of the biomimetic human organ models. The two distinct model systems have unique advantages, which assist in rapid deployment, practicality and ease of implementation for OrgOCs construction. The emergence of 3D *in vitro* cell culture approaches has therefore received widespread attention due to their high potential to overcome the limitations of conventional models. Notably, OrgOCs are promising to bridge the translational gap between biological and clinical research, enabling accelerated preclinical-to-clinical translation in medical and industrial fields.

### 2.1 OOCs

OOCs are *in vitro* human-relevant microsystems that recapitulate the structural and functional units of living organs in a microfluidic perfusion culture device (e.g., primary cells, cell lines, and stem cells). In general, the reductionist analysis of the target organ guides the design principles of OOCs construction. At first, we should understand the anatomy of the native organ in detail and design the microdevice to recapitulate the key functional unit on a chip. Then various biological analyses should be performed to identify the physiological functions in terms of cellular composition, structural organization and organ function. For instance, Ingber's team has pioneered the first lung (target organ)-on-a-chip by reconstructing the alveolar-capillary barrier (functional unit) 36. On the microchip, pulmonary alveolar epithelial cells and microvascular endothelial cells (multiple cellular compositions) can be co-cultured in the individual cell chamber by an *in vivo*-like interstitium (structural organization). The air-liquid interface with breathing motion can be simulated by integrating fluid shear stress (FSS) and cyclic stretch (CS) in the microfluidic device (organ-specific microenvironment).

OOCs can independently control or highly couple complicated microenvironmental factors, such as dynamic fluids, mechanical cues, 3D topography architecture, oxygen gradients and partitioned space, to mimic ecological niches of human native organs. These merits offered by the extracellular ecosystem can guide cell morphogenesis and the formation of functional organs. Microfluidic flow in the channels enables timely renewal of nutrients and discharge of waste, which is favorable to cell viability during long-term culture 37-39. The dynamic flow can also provide the physiological shear stress, which enhances the physiological activity of the organs and plays a crucial role in achieving systematic interaction among multiple organs within the same OOCs system 7, 40-42. Up to date, diverse micromachining technologies have been developed to fabricate biomedical OOCs systems, including photolithography, etching and 3D bioprinting 43. The mostly used photolithography process consists of several steps: 1) a photoresist coated on the substrate undergoes photochemical crosslinking and partially solidified by light exposure; 2) corrosion processing to obtain the substrate with defined structures; 3) filling the substrate with prepolymers and curing; 4) peeling off the polymer layer and integrating 44, 45. Extreme ultraviolet photolithography and electron beam lithography are usually applied to fulfill the needs of smaller channel sizes (< 10 nm) 46, 47. Typical etching is divided into wet etching and dry etching. Wet etching refers to the removal of materials in the direction of patterns under chemical etchants, while dry etching mainly uses the directional characteristics of ion bombardment and adopts reactive ion etching 43. 3D bioprinting allows the direct fabrication of objects in a layer-by-layer pattern through computer-aided manufacturing 48-50. Benefiting from various materials and high resolution, this technique has been applied to construct nearly all components (e.g, cell scaffolds, microfluidic devices and sensory connectors) of OOCs systems 43. Partitioned space can be realized within micrometer-sized channels by micromachining technologies, prompting the generation of functional interfaces/barriers (e.g., maternal-fetal interface, gas-liquid interface, gut barriers, and blood-brain barriers) 51-55. By integrating these features, a multilayer and/or multiple partition chip makes multicellular coculture *in vivo*-like possible, and helps to investigate the interaction among microorganisms, immune cells and other cells in numerous biological processes (e.g., metastasis and immune responses). 3D topography architecture can guide cell migration and differentiation in a defined orientation, such as the formation of muscle bundles, enteric tubes, vessel lumens and neural networks 56-62. In addition, mechanical motion is of the essence to mimic the physiology of motile organs, such as lungs breathing, intestines moving, heart beating, and muscle stretching exercises. It is also beneficial in maintaining organ homeostasis. Prominently, the cessation of peristalsis contributes to bacterial overgrowth and ileus and inflammatory bowel disease, consistent with pathological findings 63. The gradients of oxygen tension play an essential part in gut-microorganism interplay and enable the long-term culture of aerobic gut cells and most anaerobes. All in all, OOCs particularly emphasize manipulating cell behaviors via building human-relevant ecological niches. If necessary, electromagnetic or optical actuators can also be incorporated in the culture system of the target organ (e.g., cardiac contraction).

Significant advances in the development of OOCs have contributed to the construction of *in vivo*-like 3D organs, and these organ models can recapitulate human health and disease states for a wide spectrum of life stages, from early development to maturity 64-68. It is worth mentioning that OOCs can be employed in studying the early development of human embryos, placenta, and reproductive organs, which helps to unravel the beginning of life. Fu's group has given stem cells the ability to arrange themselves in specific structures on a chip, mimicking the process of early embryo implantation in an engineered way 66. The "mock embryo" got stuck in a 3D gel that mimics the uterine wall, forming a model of the epiblast under a constant supply of nutrients. It is surprising that the OOCs model can generate embryo-like structures with more than 90% efficiency, which is far beyond the traditional technology (less than 5%). The breakthrough highlights the importance of fluidized microchips and expands our knowledge of human embryology and reproduction. Up to now, many parts of the human body have been reproduced with OOCs, such as the brain, lung, gut, liver, kidney, islet, heart and cartilage 10, 11, 13, 69-80. These OOCs models have shown great promise in organ reconstitution, disease studies, drug screening, and so on 68, 81-85. To gain a thorough understanding of the physiological and pathological states of the human body, there has been a steady increase to establish multi-organs-on-chips in a cell type-dependent manner. Moreover, OOCs enable high-resolution *in situ* imaging, real-time monitoring of responses to external and internal stimuli on the organ level, suggesting the great potential in biomedical applications and utilizations in the foreseeable future.

### 2.2 HOs

HOs are 3D multicellular tissue constructs that originated from human pluripotent stem cells (PSCs) or adult stem cells (AdSCs) 86. They can recreate the physiological structure and function of human organs through self-assembly. In general, any PSCs-derived HOs are constructed according to the design principles involved with the sequential differentiation of stem cells. It is required to understand the germ layers the target organs are differentiated from. For instance, the human brain, heart and islet originate from ectoderm, mesoderm, and endoderm, respectively 86-90. The success of PSCs-derived HOs generation needs the timely and sequential addition of relevant factors to determine cell fate, accompanied by the self-organization of stem cells. Next, according to the developmental pathways of particular lineages, growth factors are added sequentially to establish a correct regional identity during stem cell differentiation, and organ-specific lineages in 3D HOs are identified based on exclusive biomarkers or functions. For example, human induced pluripotent stem cells (hiPSCs) can differentiate into heart organoids (target organ) by the sequential differentiation of the mesendoderm (germinal layer), the cardiac-specific mesoderm (specific direction), with B27 minus insulin (growth factors), glycogen synthase kinase 3 inhibitor CHIR99021 (growth factors) and Wnt inhibitor IWP2 (growth factors) 90. Different from PSCs-derived HOs, the formation of AdSC-derived organoids is relatively simple, which doesn't need to guide it through the germ layers 86. Usually, we need to isolate tissue-specific stem cell populations from adult tissues. Then these stem cell populations are implanted in ECM and engendered in combination with particular tissue development components. Back in 2009, Hans Clevers's team first implemented Lgr5^+^ stem cells-derived intestinal organoids isolated from murine small intestine, containing a central lumen lined by villus-like epithelium and surrounding crypt-like domains 84. Exogenous factors such as Wnt agonist R-spondin 1, epidermal growth factor, transgenic expression of Noggin as well as ECM component are pivotal requirements for intestinal epithelial growth and region-specific formation 84. In 2011, this team, for the first time, adapted the culture conditions and established human epithelial organoids from the human colon, Adenoma, Adenocarcinoma and Barrett's epithelium 91. Overall, these two different HOs paradigms have their own advantages and disadvantages. PSCs-derived organoids require longer induction cycles and do not support passage cultivation. AdSC-derived organoids from patient tissues enable the formation of an isogenic tissue having identical genes, potentially paving the path towards patient-specific therapies and personalized medicine 25. Unfortunately, organoids derived from tissue biopsies are often limited in their ability to differentiate and generally contain only certain parts of the organ 92. For instance, intestinal stem cells from adults will only generate intestinal epithelial cells, nothing more. In contrast, PSCs-derived organoids allow for the forming of more complex structures such as vascularization and immune systems by integrating multicellular elements. Besides, AdSC-derived organoids lack long-term stability due to individual differences and high heterogeneity 86. For decades, stem cells and developmental biology have offered a strong foundation upon which researchers can recreate the key aspects of organogenesis *in vitro*. HOs systems, as a versatile tool, show significant promise for a wide spectrum of biomedical applications in studying organ development, tissue regeneration, disease occurrence, drug screening and so on 93-97. So far, various HOs have sprung up worldwide, covering the human brain, cortex, eye, gut, lung, heart, liver, kidney, islet and other parts 78, 98-106. The main sources for generating HOs are stem cells from cancer biopsy or surgery. It is worth mentioning that HOs derived from patients can faithfully reflect the characteristics of the original cancer. Moreover, these cancer models have a robust expansion ability and thereby can be stably cultured and passaged. In comparison to traditional cell lines, HOs can preserve cell phenotype and genotype for a long term *in vitro*, and reveal common recurrent genetic lesions 26, 38, 107. HOs can be exploited to investigate many different aspects of tumors (early, middle, and late stages), including homeostasis, tumorigenesis, cancer transformation, and relevant mechanisms 108-112. This undoubtedly prompted scientists to start a race against cancer, hoping to solve the long-standing mysteries of cancer. The breakthrough technology may greatly contribute to improving the current diagnostic and therapeutic agents, improving the success rate of drug development, as well as optimizing the clinical usage of medications.

Every coin has two sides. Conventional culture techniques afford a simple and effective approach to generating organoids, and the technical threshold is relatively low for most laboratories. Unfortunately, their operational simplicity often comes at the expense of precise control. In contrast to the highly reproducible process of organogenesis *in vivo*, HOs models often display high heterogeneity in tissue size, structure, and function, which limits their applications to some extent 29, 113, 114. Moreover, the conventional approaches highly rely on animal tissue-derived 3D ECM with undefined configurations, poor reproducibility, and high heterogeneity 107, 115. On one hand, many efforts have been made to replace uncertain substrates with biological scaffold materials such as natural or synthetic hydrogels with given concentration, adjustable stiffness and tunable morphology 116-120. On the other hand, engineering technologies (e.g., micro-column/well chips and commercialized grooved plates) have been exploited to avoid using ECM 121-124. Besides, most models lack blood vascular and the immune system, failing to faithfully reflect human biology 17, 107. Usually, vascularized human organoids are produced by the physical mixing of organoids and endothelial cells, and it is difficult to form vascularized organoids in a physiological context 33, 125, 126. As mentioned above, these limitations may hinder the manufacture of more representative human organoids in a precise way, and a deeper understanding of human physiology and pathophysiology as well. A combination of OOCs technologies is expected to solve these bottlenecks, and breakthroughs and meaningful achievements are on the way.

### 2.3 OrgOCs

Theoretically, OrgOCs are highly biomimetic *in vitro* models by combining two cutting-edge technologies (HOs and OOCs) in the field of life science and engineering. In 2019, the concept of OrgOCs was proposed firstly in Science, which refers to an "upgraded version" or an extension of OOCs. Nowadays, OrgOCs have been widely used in the fields of drug development, disease modeling and precision medicine 2, 127-131. Generally, it is a highly technical and multidisciplinary industry, with a developmental procedure ranging from chip design and fabrication, model establishment and functional evaluation, and finally to biological study or drug testing. The construction of the OrgOCs needs to follow the specific principles of organ developmental biology and take the feasibility and stability of the microdevice into account. The first step is to confirm cell types and space distribution and design the microstructures in the chip accordingly. Then, related biochemical microenvironmental elements should be incorporated into the system to induce cellular behaviors according to the developmental pathways of particular lineages. Finally, it is necessary to optimize the OrgOCs system by manufacturing the organ with desired structural and functional features. Together, as an extension in biotechnology, OrgOC is a collaborative technology of OOCs and HOs that can potentially address the theoretical and technical bottlenecks of traditional approaches.

The aforementioned high heterogeneity has been known as a major issue that limits the applications of HOs models. Recent advances in OrgOCs technology suggest various methods to tackle this problem using microengineered culture devices. A series of OrgOCs with microwell structure provides an instructive cue to guide the formation of HOs with uniform size and reduced stochasticity and variability, potentially reducing the unreliability of experimental results caused by heterogeneity 30, 90, 114, 132. Considering the uniformed size, controllability and modularity, this strategy is adjustable for size-based selection and also integrate into other systems for improved homogeneity of the organoid populations. Researchers have innovatively combined droplet microfluidic technology and wet spinning with OrgOCs, allowing for the in situ encapsulation and formation of functional human organoids with features of uniformed 3D structure and high throughput testing 113-135. At present, many commercial culture plates (plastic and vitreous forms) have been widely used for 3D organoid formation and subsequent drug testing 136-138. Many other efforts have also been made by integrating sensing elements into culture platforms for continuous monitoring, screening and analysis of organoids. Besides, the emerging 3D bioprinting technology drives the high-throughput and high-precision construction of OrgOCs. Also, artificial intelligence (AI), multi-organs, computer science, multiplex biosensors, multi-omics analysis, and gene editing can be incorporated into OrgOCs systems to improve the real-time detection and analysis of biological signals in a compositive manner. Prominently, OrgOCs technology can significantly increase reliability in the prediction of drug efficacy and safety, while reducing the cost and drug failure rate, potentially providing strong scientific evidence for clinical trials. These features are not available with conventional 2D cell culture and animal models, where there are obvious mismatches between the data derived from animal and human, or from *in vitro* static and *in vivo* dynamic conditions 38, 54, 139.

Overall, the disruptive OrgOCs technology can not only recapitulate human physiology to a large extent but also forecast the body's response to drugs and different stimuli. Although the advanced OrgOCs systems are still far from faithfully recreating the functionality of native human organs, it has already demonstrated the unique advantages of simulating key aspects of human physiology and pathophysiology. Their unique features make them a reliable platform to reduce or even replace animal experiments for the preclinical assessment of drugs 140-144. Even so, there is still a lot of room for improvement in OrgOCs models, and we can expect that these models offer increasing possibilities to reproduce near-physiological complex tissues and organs.

## 3. Progress of OrgOCs in biomedical applications

In recent years, significant progress has been made in the field of biomedicine using self-assembled and engineered 3D OrgOCs models *in vitro*. In the following sections, we outline recent advances in OrgOCs models for biological study, disease modeling and precision medicine. The summary of the comparisons of different model systems for biomedicine can be seen in **Table 1**. In addition, we have provided a summary of the key elements (e.g., types of organoids-on-chips, organoid resources, co-culture cells, ECM and chip design) for the construction of OrgOCs models as well as the biomedical applications, important findings and unique advantages of existing OrgOCs models (**Table 2**).

### 3.1 Biological study

#### 3.1.1 Organ development

Human organ development is precisely modulated by many physicochemical, biological and mechanical factors, and these factors could be recreated to construct artificial living organ systems *in vitro*. OrgOCs have been widely used to model organ development by actuating stem cells behaviors and integrating cell ecological niches with the development of engineering and cell biology.

**Figure 2:**
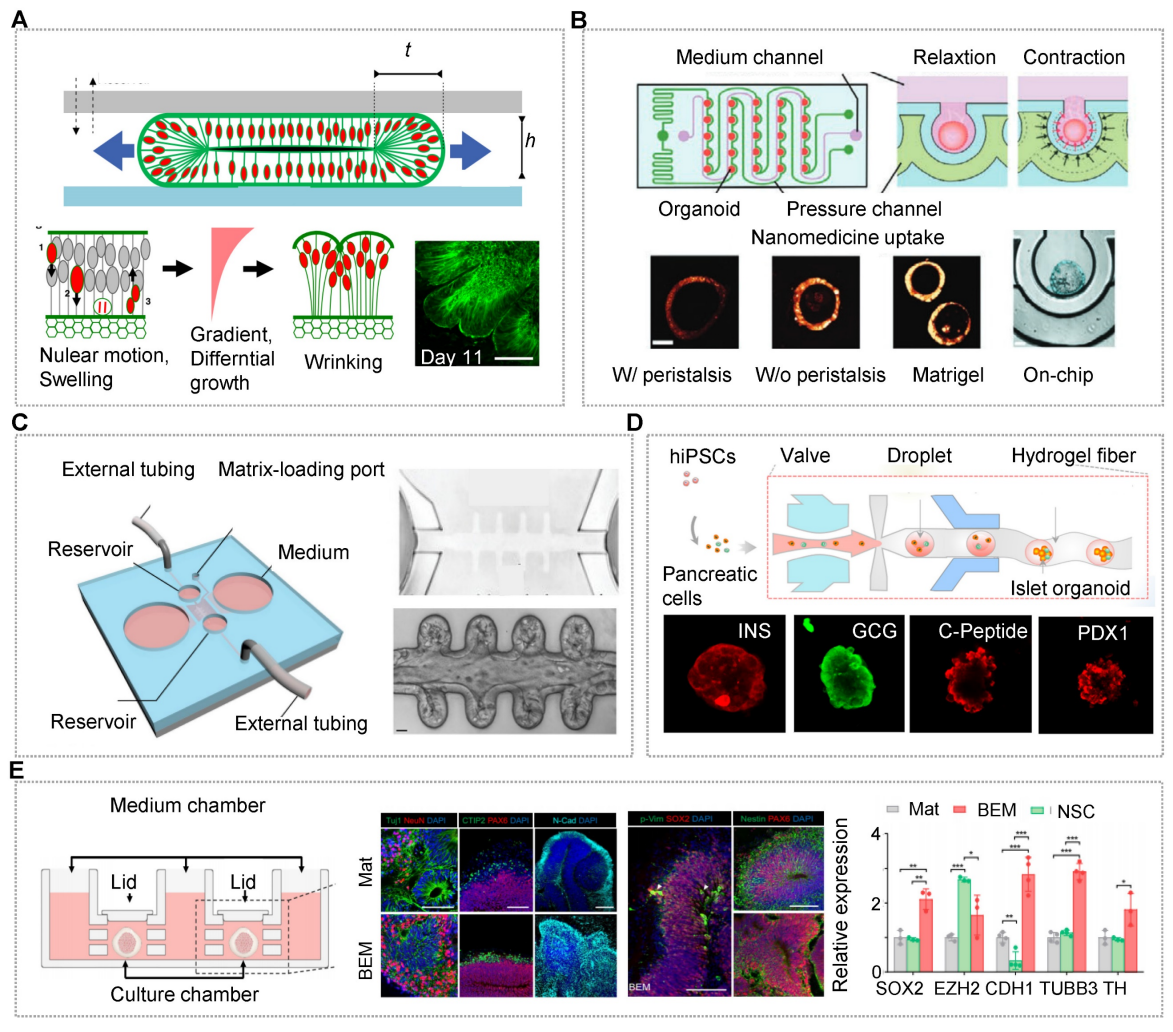
OrgOCs platform for human organ development in biological study. (A) Human brain organoids-on-a-chip revealed the physical mechanisms and intrinsic cell behavior during brain development. Adapted with permission 165. Copyright 2019 MDPI. (B) Human colon tumor organoids-on-a-chip system with a representative *in vivo*-like curved morphology and peristalsis traits. Adapted with permission 16. Copyright 2023 Wiley-VCH GmbH. (C) A novel chip with crypts-like microchannels to induce intestinal organoids morphogenesis, including perfusable mini-gut tubes, a near-physiological spatial arrangement of a gut lumen, crypt-like domains and villus-like constructions. This work demonstrates the importance of spatial confinement in organ development. Adapted with permission 16. Copyright 2023 Wiley-VCH GmbH. (D) Microfluidic-based 3D carriers assisted in the differentiation and maturity of islet organoids under more physiological microenvironments. Adapted with permission 133. Copyright 2021 American Chemical Society. (E) In OrgOCs reactors, fluid could promote cell proliferation and reduce cell apoptosis in brain organoids. Adapted with permission 146. Copyright 2021 Springer Nature.

The OrgOCs platform contributes to discovering the underlying physical mechanisms and the intrinsic cell behavior during the organ development. Reiner's group has modelled the physics of the folding human brain organoids on a microchip, presenting temporal dynamics similar to MRI images of fetal brains 145. The findings reveal that mechanical instability existed in brain development, in which cytoskeletal contraction and nuclear motion contend against each other to balance the differential growth of organoids (Figure 2A) 165. Except for this intrinsic mechanical factor, the artificial motion is also crucial to maintain organ physiological functions. Jin's group, for the first time, developed a gastrointestinal organoid-on-a-chip system with a representative *in vivo*-like curved morphology and peristalsis traits 124. The OrgOC is composed of microwell arrays for human colon tumor organoid growth, and surrounding pressure channels for the control of peristalsis amplitude and cycle to mimic the natural rhythm *in vivo*. Notably, the proliferation of Lgr5^+^ cells and the expression of Ki67 with the *in vivo*-like natural rhythm can be up-regulated 1.6-fold and 2.1-fold in the colon tumor organoids, compared with the traditional model 124. Moreover, mechanical stimuli have impacted the drug uptake in the organoids, revealing the importance of mechanical factors in both organ development and drug assessment (Figure 2B) 16.

Among many elements, spatial confinement is an essential one, which affects the biomanufacturing processes, and more importantly, guides biological behaviors of organoids at multiscale levels 126. Lutolf's group exploited a novel chip with crypt-like microchannels to model intestinal organoid morphogenesis 57. In the OrgOCs model, intestinal stem cells can efficiently form perfusable mini-gut tubes with an in vivo-like spatial arrangement of a gut lumen, crypt-like domains and villus-like constructions to that in the human body (Figure 2C) 16. This spatial self-organization maintained organ lifespan and homeostasis, which avoided the limitations of regular organoids culture models in a stochastically developing manner. Similarly, geometric constraints such as 3D topology can be harnessed to promote *in vitro* stem cell self-organization along predefined spatial boundaries and significantly drive deterministic organoid formation 62. Different from 3D self-assembly models, organoids also can be broken into single cells or small clusters and then laid flat on the microchannels, generating 3D intestinal/pulmonary organs with typical villus and functional barriers 103, 151, 166-168.

This tactic helps to study barrier-based functional identification, such as drug absorption and penetration. Besides, 3D carriers (e.g., microgels, capsules and fibers) with confirmed spatial confinement can regulate the evolutionary dynamics of living systems during organ development 44, 113, 133, 134, 169. For islet organoids within a 3D fiber carrier, the expression of related genes increased remarkably in comparison to that in conventional cultures 133. The finding indicated that 3D models assist in the differentiation and maturation of islet organoids with *in vivo*-like microenvironments (Figure 2D).

Dynamic flow by extra injection pumps or pump-free rotators can easily be introduced in microchannels of OrgOCs devices to enhance the physiological relevance of 3D organ. Kim's group confirmed intestinal 3D villi-like morphogenesis occurred under fluid flow conditions, which did not occur when basal flow was ceased 152. Fundamentally, microfluidic periodic flow can take away metabolic waste, allow a constant infusion of oxygen and renew nutrients, avoiding necrosis in the inner core of the organoids, and extending their lifespans to a fully mature *in vitro* model. In OrgOC reactors designed by Cho's group, a dynamic environment was found to greatly promote cell proliferation and reduce cell apoptosis in brain organoids compared to static culture (Figure 2E) 146. Moreover, during development *in vitro*, the functionality of organoids largely depends on their maturity and complexity. Deng et al. reported an impressive OrgOCs platform to study placental development for the first time. In the report, the dynamic flow enhanced the expression of trophoblast progenitor markers and promoted multivarious subtypes differentiation in the placental trophoblast-like organoids 39. Human islet organoids generated by Tao et al. contained classical endocrine cells (e.g., pancreatic α-, β-, γ-, and δ-cells) with regulating functions of blood glucose homeostasis under a perfused OrgOC platform analogous to human pancreatic islets. Significantly, compared to static models, islet organoids exhibited much stronger pancreatic-related signals, including enhanced insulin secretion and Ca^2+^ flux. These data highlighted the essential role of dynamic flow in improving the physiological relevance of human-engineered islet models. Similar reports have also proved that dynamic fluids can enhance the specific organic lineages during the development of organoids (e.g., brain, liver, kidney and placenta) 35, 72, 121, 170. These studies exemplify how OOCs and HOs can be synergistically fabricated to achieve maximal physiological maturation not available when either technology is used alone.

Despite great efforts, there is still a long way to fully recapitulate native human organs owing to the highly complex microenvironment and tissue-tissue interplay in terms of mature organ architectures and functions. Such a gap also poses a huge challenge for OrgOCs to advance the preclinical-to-clinical transition in biomedical and industrial fields. Hence, it is highly desirable to establish high-fidelity organoids with key characteristics of mature organs under both physiological and pathological conditions. The essential cues offered by the extracellular matrix (ECM) that render the biocompatibility and bioactivity, can mediate the cellular behaviors and functions of certain tissues or organs. Typical animal-derived ECM (e.g., Matrigel and decellularized ECM) are often used in the OrgOC device to explore the effects on organ development. In the OrgOC platform, decellularized ECM from human brains can provide brain-specific cues for enhanced neurogenesis, the formation of larger neuroepithelial structures, and the organization of layered cortex (Figure 2E) 146. Kim et al. found that gastrointestinal organoids cultured in the tissue-derived extracellular matrix displayed similar gene profiles to those of the native tissues 115. In addition, the tissue-derived extracellular matrix provided a microenvironment that was conducive to the long-term subculture and transplantation of organoids. Usually, this type of ECM often contains complex compositions (polysaccharides, proteins, and growth factors) and uncertain concentrations due to the particular source. In addition, ill-defined matrices ignore the important role of individual ECM factors in recapitulating key events in organogenesis and reorganization 107, 115, 171. In the context, Ranga et al. successfully decoupled the regulatory role of most factors (e.g., laminin, entactin, collagen, perlecan, and fibronectin) in Matrigel on stem cell fate decision (e.g., proliferation and differentiation) and neural tube morphogenesis (e.g., apical-basal polarity and phenotype identify) 172. Notably, laminin was the most vital modulator among the tested ECM ingredients. Now researchers are trying to figure out the alternatives of ECM for engineering stem cell organoids *in vitro*. Typically, hydrogel materials such as polyethylene glycol, gelatin, and alginate have been developed and applied in microenvironment creation, near-physiological organ formation, pre-vascularization before organoid transplantation, and even therapeutic modality development. For example, Spence's team demonstrated that alginate could support human intestinal organoid amplification, differentiation, and maturation *in vivo*, which was indistinguishable from organoids grown in Matrigel 119. Lutolf's team found that intestinal stem cell activity and proliferation required a fibronectin-based adhesion. Laminin-based adhesion contributed to stem cell differentiation and organoid formation. Kumacheva and coworkers demonstrated that patient-derived breast tumor organoids developed in gelatin hydrogels have similar morphological traits, gene expression profiles, and drug responses to those of their native tumors and organoids grown in decellularized ECM 173. Rossen et al. presented a sacrificial alginate scaffold strategy to generate therapeutic organoids 118. Markedly, intramuscular injections of the vascular units could quickly restore vascular perfusion after injecting the organoids within 1 week in a mouse model of peripheral artery disease 118. Indeed, burgeoning biomaterials with defined composition and tunable properties are suitable substitutes to mimic native 3D matrices and steer organoid morphogenesis and maturation by spatiotemporal control over microenvironmental cues.

Except for the explicit ingredients, ECM stiffness also varies within the microenvironment, affecting human organ development. For example, the shear modulus of the healthy human endometrium was assessed to be higher during the proliferative phase (3.34±0.42 kPa) compared to the early secretory phase (1.97±0.34 kPa) 174. Indeed, it is well-known that lower stiffness aids the implantation of the human embryo. Softer ECM can directly affect key events during early pregnancy by enhancing the decidualization of endometrial stromal cells, promoting blastocyst invasion, and impacting embryo-endometrial interaction 174. Lutolf's group found that high matrix stiffness greatly heightened stem cell expansion via a Yes-associated protein 1 (YAP1)-dependent signal pathway, subsequently impacting the differentiation and formation of intestinal organoids 173. Moreover, niche hardness also leads to the human organs being in a pathological state, such as liver cirrhosis, infertility, scleroderma and paralysis 171, 175-180. In another work, Anseth's Lab applied allyl sulfide hydrogels to precisely control the matrix environment of intestinal organoids by changing the degree of degradation in a spatiotemporal manner 181. The results demonstrated that colony survival in dynamic hydrogels was dependent on matrix moduli and that crypt formation, size, and number per colony were functions of matrix softening. In addition to these, one physiological activity often involves the fine regulation of ECM, such as angiogenesis. Soft matrices are known to aid angiogenesis, however, stiff matrices serve as a strong scaffold for angiogenesis, as sprouts align along a VEGF gradient more readily in stiff than in soft ECM 126. So, in the design principles of OrgOCs, building artificial scaffolds or a well-defined matrix is a reasonable element for organoid development.

In addition to those mentioned above, biochemical factors, oxygen gradients, electrical signals, and optical stimuli also play key roles in organ development 158, 182-191. *In vivo*, the formation of the body axis involves morphogenic gradients, such as rostro-caudal and dorso-ventral axes that allow the differentiation and organization of heterogeneous brain architectures with different regions along the axis 17, 192, 193. For instance, a sonic hedgehog protein gradient could specify distinct dorso-ventral and antero-posterior positional domains in developing forebrain organoids 192. Therefore, the generation of factor gradients is critical for guiding heterogeneous tissues with spatial topography. Microfluidic devices can be employed to create a gradient by sequential diffusion of input factors 17. Based on this principle, Kirkeby's group established a WNT signaling gradient on a chip to model early neural tube development. Interestingly, the proposed on-chip regionalization culture system exhibited progressive caudalization from the forebrain to the midbrain to the hindbrain along the rostro-caudal axis 193. Oxygen gradient is also a factor that leads to cellular spatial patterning during development. *In vivo*, a self-sustaining oxygen gradient exists in the human gut-microbiome interface 44, 68. On one hand, this oxygen difference remains obligate anaerobe vitality and provides a stable symbiotic environment. On the other hand, a physiological oxygen gradient formed along the length of crypts enhances stem-cell proliferation and facilitates cell compartmentalization at the crypt base 185. Besides, oxygen tension also directs the trophoblast differentiation pathway. Notably, low oxygen could inhibit the differentiation of human stem cell-derived primary cytotrophoblasts (CTBs) into syncytiotrophoblasts and promote the initial differentiation of CTBs into HLA-G^+^ extravillous trophoblasts 194, 195. Other microenvironmental factors (e.g., electrical signals and optical stimuli) are also involved in regulating cell behaviors during development, such as neuronal activity and muscle contraction 196-199. Quadrato et al. found that sensory stimulation with light could modulate stem cells-derived brain organoids to develop spontaneous active neuronal networks and photosensitive neurons 196. Notably, the activity-dependent gene FOS was upregulated in eight-month old organoids after light exposure. Overall, the emerging OrgOCs technology helps to enhance the physiological relevance of organoids to real organs and better understand the development of the body in a sophisticated and more reliable *in vitro* platform.

#### 3.1.2 Vascularization

The microvascular system is a continuous conduit to deliver nutrients, oxygen, waste products, immune cells and CO_2_, and couples the fate of all organs and their functions by inter-organ interplay. *In vivo*, tissue and organ development is tightly related to vasculature from embryonic development at the early stage. *In vitro*, tissue is vascularized through vasculogenesis or angiogenesis 33, 88, 200-203. Correspondingly, vasculogenesis-on-a-chip models can be created by mixing endothelial cells and fibroblasts or mesenchymal stem cells into a gel channel on a chip, forming *in vivo*-like capillaries by self-organization 77, 126, 204, 205. Angiogenesis-on-a-chip models are built with angiogenic sprouts of fibroblast spheroids guided from endothelial cells, where these cells are inoculated on the spatially separated channels 206-208. These are the most basic and simplest methods to reconstruct a self-organized microvascular network of organs *in vivo*. At present, a series of functional organ barriers with vascularization features have been reproduced, including the blood-brain barrier, placental barrier and capillary vessel barrier 38, 51, 53, 209, 210. Not limited to these, a real sense of engineered vascularized organs, that are vascularized organoids, are formed following the same strategy.

**Figure 3:**
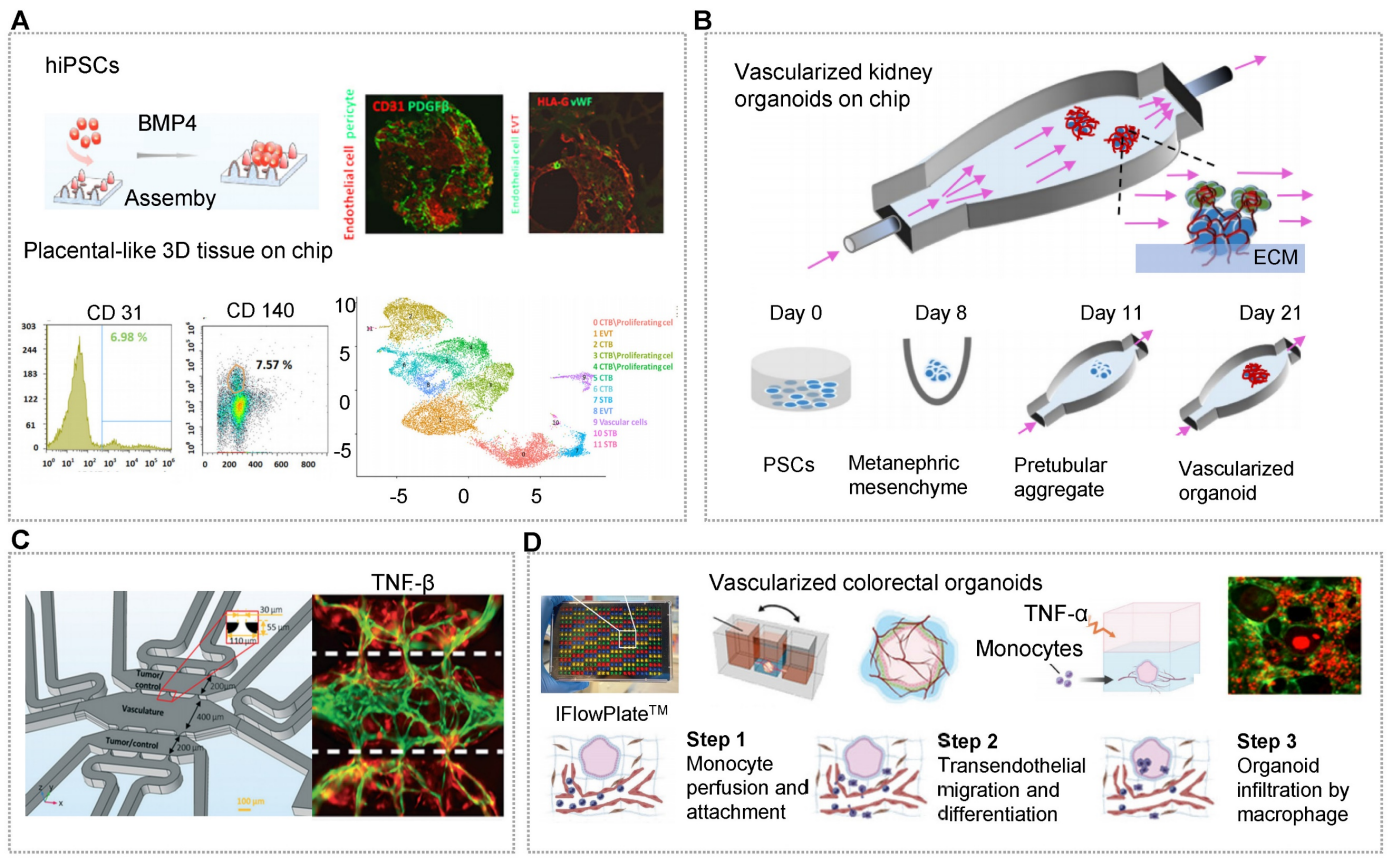
OrgOCs platform for vascularization in biological study. (A) The formation of vascularized placental-like organoids resembled first-trimester human placental development in a microfluidic chip platform. Adapted with permission 30. Copyright 2022 Wiley Periodicals LLC on behalf of American Institute of Chemical Engineers. (B) Interstitial flows could expand the endogenous pool of endothelial progenitor cells and enhance the vascularization and maturation of kidney organoids derived from hiPSCs. Adapted with permission 212. Copyright 2021 MDPI. (C) Implanting the functional neurovascular cerebral organoids on a chip induced angiogenesis by co-culturing adscititious endothelial cells. Adapted with permission 61. Copyright 2022 The Royal Society of Chemistry. (D) Organoids with a perfused microvasculature reconstituted the process of monocyte infiltration into colon organoids in the circulatory system using customizable IFlowPlates. Adapted with permission 137. Copyright 2020 Wiley-VCH GmbH.

Usually, vascularized organoids can be generated containing endogenous vascular cells by adding indispensable growth factors (e.g., VEGFA) during induction stages 30, 33, 126, 162, 211. Qin's group presented a novel strategy to engineer placenta-like organoids containing endogenous vascular cells (endothelial cells and pericytes) from hiPSCs on micropillar chips (Figure 3A) 30. These vascularized placental-like organoids resemble first-trimester human placental development in terms of complex cellular components, placental villous-like structure, and placental-specific hormone secretory function 30. Unfortunately, there is no perfusable vascular network in these organoid tissues. Indeed, most organoids derived from hiPSCs have organ-specific and tubular compartments that are largely avascular and/or immature. Even so, the microsystem demonstrated the morphological and physiological correlation of the human placenta. On the same microfluidic chips, Morizane and his coworker applied interstitial flows to expand the endogenous pool of endothelial progenitor cells and enhance vascularization and maturation of kidney organoids from hiPSCs 77. These kidney organoids contained vascular networks with perfusable lumens surrounded by mural cells. The vascularized kidney organoids can mirror the early stages of development with features of glomerular vascularization and maturation during nephrogenesis (Figure 3B) 212. OrgOCs platforms with continuous long-term perfusion can potentially overcome major limitations of traditional technology that hinder their broader applications.

In addition to inducing stem cells to produce organoids with endogenous cellular components, it is also possible to design multicompartments to recapitulate complex stratified 3D organs with endogenous stromal components, thus surpassing some of the limitations of traditional culture systems. One design principle of vascularized organoid-on-a-chip is to implant the functional organoids on a chip and then induce angiogenesis via adscititious endothelial cells. Ranga's group developed a co-differentiated approach to generate neurovascular cerebral organoids which showed vascular cells sprout, significant expansion and vascular network consolidation 61. With the presence of the vascular cells (endothelial cells and pericytes), the gene expression of immature and deep layer neurons were 2.7 folds and 5.5 folds lower respectively in cerebral organoids than that in mono organoids mode. Strikingly, vascular cells-related markers were highly upregulated (Figure 3C) 61. Likewise, emulating vascularized patient-derived tumor organoids by incorporating tumor organoids into microvascular beds contributes to the advancement in tumor progression and cancer therapeutics. Steven's group has confirmed the angiogenic potential of patient-derived tumor organoids in a three-chambered OrgOCs system, displaying the enhanced growth and extension of vessels than that in the control group 116. A typical penetrating vascular network can be observed from 6-days to 22-days in a stable and expandable manner. Specifically, the migration of the tumor into the microvascular chamber was promoted by delivering TGFβ through the vascular network. Moreover, the interactions of basement membrane, vascular endothelium and other cell types through paracrine signaling can further promote the vascularization of organoid models. Of course, vascularized organoids can also be formed via physical blending and self-assembly of the organoids and endothelial cells 162.

Signally, a perfused vascular network allows for the transport of cells and nutrients as well as the removal of waste. Despite this tremendous progress, this area is still in its infancy to achieve standard vessel networks in organoids similar to those *in vivo*. The majority of vascular networks are not adequately elaborate to simulate signals, such as the transport of immune cells and blood cells through vascular networks to targeted locations. Using customizable IFlowPlates, Zhang's group successfully introduced monocytes into the vascularized colon organoids, reconstituting the process of monocyte infiltration into colon organoids in the circulatory system, including monocyte perfusion and attachment, transendothelial migration and differentiation, and organoid infiltration by macrophage in response to tumor necrosis factor (TNF-α) stimulation 137. Surprisingly, macrophages were capable of infiltrating nearly 80% of colon organoids after TNF-α stimulation for 1 day (Figure 3D). Furthermore, the suitable regional organization and interaction between multi-organs can be constructed to simulate *in vivo* physiology and functionality. Although some successes in forming single vascularized organoids with an OrgOC platform have been reported, we argue that the vascularized organoid models facilitate to create multi-organoid systems featured with tissue-specific microvasculature through a dynamic vascular network. Zhang and Radisic et al. designed a versatile 96-well plate combining the 3D stamping technique with biomaterials, in which a central tube channel is used for supporting a perfusable vascular system and self-organization of various parenchymal tissues 213. Uniquely, the systematic microphysiological model connected two or more tissues (vascularized cardiac, hepatic, breast cancer, and pancreatic cancer organoids) compartments through a common vasculature, contributing to the advance in cancer treatment and research.

It is well-known that vasculature is key to organ development, and the absence of vasculature is observed to greatly limit the generation of HOs 213, 214. Representation of an *in vivo*-like vasculature using OrgOC platforms contributes to organoid growth and formation for biological and pathological studies as well as drug screening 129, 137, 215. However, the formation of a complex network of blood vessels is often limited due to the limited size and space of the chip. More broadly, the design of customizable OrgOC devices with consumer-grade 3D printers and biocompatible materials may pave the way to produce *in vitro* vascularized tissues in a precise and reproducible manner at a macro-scale level 61, 204, 216. With the development of new technology, it is expected to solve long-term bottlenecks in how to form 3D vascularized organoids with comparable size to human organs, which is fundamental for transplants. We believe that the vasculature-linked, phenotypically stable human organoids can be efficiently constructed, which may broaden the clinical applications of tissue chips.

#### 3.1.3 Host-immune response

**Figure 4:**
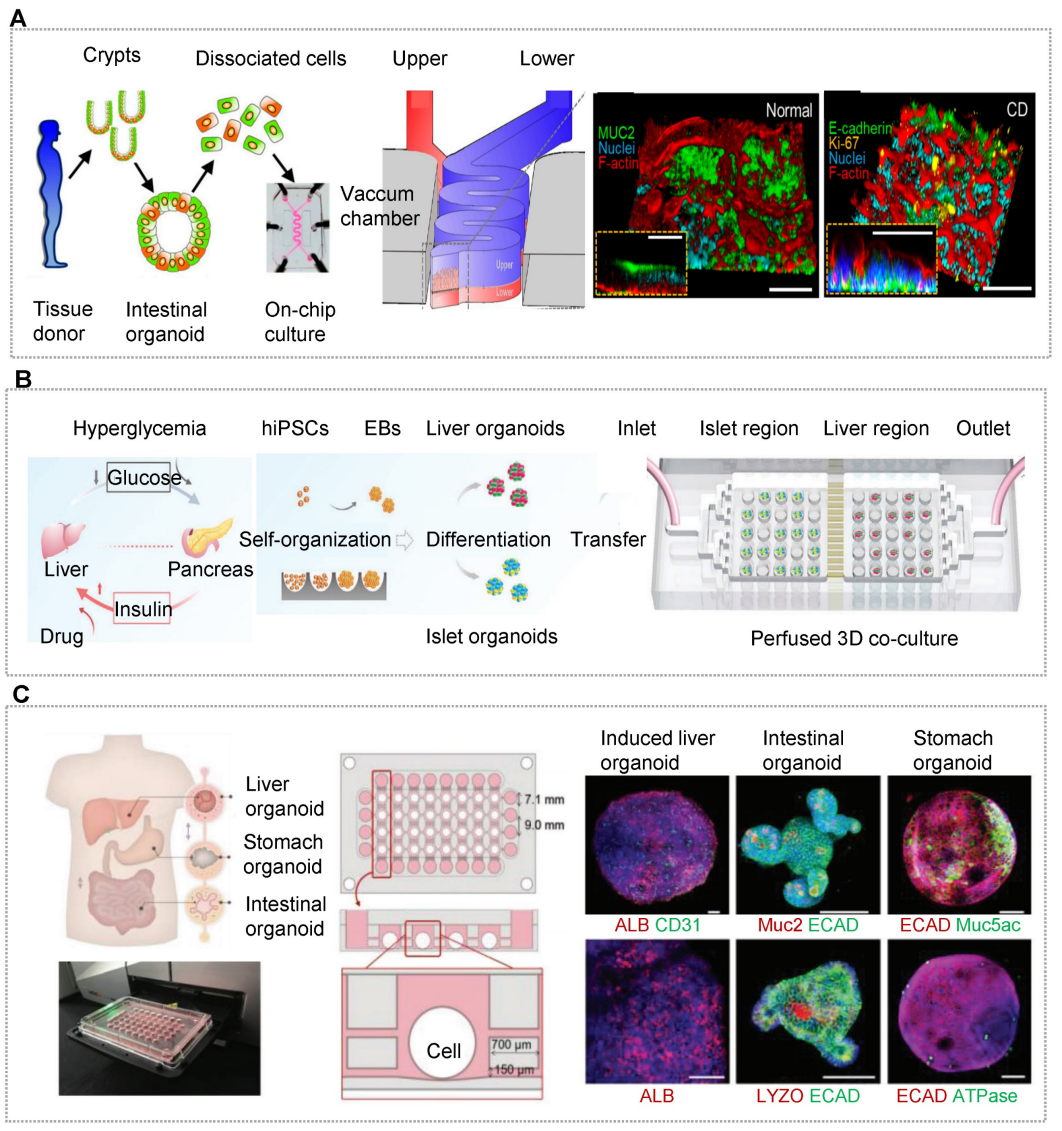
OrgOCs platform for exploring inter-organ communication in biological study. (A) A colon biopsies-derived intestinal organoid chip showed the potential capacity of human milk oligosaccharides in modulating immune function and the gut barrier. Adapted with permission 155. Copyright 2020 MDPI. (B) Multi-organoids-on-a-chip platform recapitulated human liver-islet axis in a circulatory perfusion system. Adapted with permission 10. Copyright 2021 Wiley-VCH GmbH. (C) A series-wound liver, stomach, and intestinal organoids model in a high-throughput microfluidic setup was applied to assess drug metabolism and bile acid-induced regulation. Adapted with permission 162. Copyright 2018 WILEY-VCH Verlag GmbH & Co. KGaA, Weinheim.

Recently, one of the major paradigms shifts in medicine relates to the discovery of the gut microbiome's role in human health and diseases. Moreover, accumulating evidence indicates that intestinal epithelium and other cell types participate in the complex crosstalk between gut microbiome and host immunity 57, 75, 217-223. Benefiting from the advantages of OOCs, these elements can be incorporated into one microchip. Using a human gut-on-a-chip microsystem, Kim's team found that intestinal barrier impairment orchestrated the onset of inflammatory host-microbiome communication 52. The co-cultured immune cells and dextran sodium sulfate-sensitized epithelium can exacerbate the oxidative stress, in which the luminal microbial stimulation causes the secretion of inflammatory factors and the recruitment of immune cells. By contrast, an intact intestinal barrier with immune elements can dramatically elevate oxidative stress and inflammatory factor secretion in response to LPS and nonpathogenic *E. coli* at the physiological level. Probiotic treatment, which is thought to facilitate intestinal homeostasis, is found to reduce oxidative stress, but fails to restore barrier impairment and inhibit proinflammation 52. To better model human gut-microbiome interplay, Sasan et al. co-cultured intestinal cells from normal human ileum, and anaerobic/aerobic human commensal gut microbiota from human infant stool specimens 219. Indeed, compared to adult human-derived stool, lower bacterial richness was observed in the infant stool stock on a chip. In another work, Shin et al. formed intestinal organoids derived from the patients with Crohn's disease, ulcerative colitis and colorectal cancer (Figure 4A) 155. Notably, co-culture with the human fecal microbiome on chip in anoxic-oxic interface resulted in the formation of stochastic microcolonies without a loss of epithelial barrier function. Furthermore, oxygen sensors and trans-epithelial electrical resistance meters were induced into the OOCs for dynamic monitoring of intestinal barrier function with multiple cell types and individual bacterial strains. In the gut-microbiota ecosystem, Juge's group examined the effect of human milk oligosaccharides (HMOs) on gut microbiome on an intestinal organoid-derived microfluidic chip and illustrated the potential capacity of HMOs in modulating immune function and gut barrier in adults 153. The study demonstrated that fermented 2'-O-fucosyllactose (2'FL), lacto-N-neotetraose, and combinations induce the number of bifidobacteria, accompanied by accumulated short-chain fatty acid, in particular butyrate with 2'FL. Besides, all of the human intestinal organoids taken from proximal, transverse, and distal colon biopsies showed upregulated claudin-5 by fermented 2'FL treatment, supporting the benefits of HMOs on human health 153. Beebe's group presented an *in vitro* microsystem that investigated both physical contact and dynamic communication between the host and pathogens at the human terminal bronchi 224. The model detected varied immune responses to the ΔlaeA fungal mutant in monomicrobial and polymicrobial cultures. The differences in inflammatory cytokine production and recruitment of mononuclear phagocytes indicated the complex host-microbiome crosstalk. Of particular note, the crosstalk was altered with direct contact with *P. aeruginosa*, implying the high complexity of the human microbiome and infectious diseases.

In addition to microorganism, immunosurveillance of the gastrointestinal epithelium by immune cells (such as MNPs) also participates in the host-immune response, which is crucial in maintaining intestine homeostasis. Nevertheless, it is difficult to explore the interactions between human gastrointestinal epithelium and immune cells with conventional cell cultures and animals, as these models fail to fully represent human tissues. Obviously, OrgOC can serve as a reliable alternative for these studies. For example, Michelle et al. reproduced physiological interactions between dendritic cells and epithelial cells derived from gastric organoids using a gut organoid chip 116. Besides, a colon inflammation model was constructed with the innate immune function by simply circulating monocytes through the vasculature which can emulate the exact process that happens in the body without the need to activate the monocytes with M-CSF 137. Circulating monocytes were differentiated into macrophages, and observed to be recruited from the vasculature and infiltrate the colon organoids in response to TNF stimulation. This work demonstrated the tight relation of monocyte recruitment to inflammation and TNF-α release. Moreover, other cell types also influence the microbiome-host immunity system via the gut-organ axis 225. For example, the brain regulates the secretion of signaling factors from cells in the gastrointestinal tract, affecting intestinal motility, microbiota composition, and mucosal immune response 225. In recent years, there has been increasing evidence that the gut microbiota-host immune system has an impact on the physiological and pathological functions of other organs as well 226, 227. Certainly, the interaction between the gut/microbiome and other organs is a two-way communication 225, 228. For instance, as a vital participant, the microbiome and its metabolic products, including short-chain fatty acids (SCFA), directly and indirectly affect the broader gut-immune-liver-brain axis 229. Griffith and coworkers developed a Transwell-based microphysiological system composed of the gut, liver and brain with integrated fluid channels 229. In the presence and absence of circulating T cells and T helper cells, the concentration of inflammatory cytokines were reduced by microbiome-derived short-chain fatty acids (SCFA). At the same time, SCFA led to the enrichment of pathology-related pathways and increased expression of genes related to neurodegenerative pathology 229. In another example, microbial metabolism can produce uremic toxins such as trimethylamine-n-oxide, p-cresol sulfate, and indole sulfate 228. In turn, uremia disrupts gut microbiota composition and metabolism. Any interference between this two-way communication can lead to a variety of serious complications such as chronic kidney disease, end-stage kidney disease, and septic acute kidney injury 228.

Although studying the host-immune response using OrgOCs are still in their infancy, we believe the development of OOCs will pave the way for developing OrgOCs. Additionally, OOCs can contribute to studying the host-microbiome interactions in other organs (e.g., brain, lung, amnion and placenta) 26, 30, 230-232. At present, the long-term culture of human organoids and microbial on microchips is difficult, and we expect that the cross-fuse of multiple technologies can help the system to quickly reassemble and comprehensively parse the interplay. Indeed, it is not out of the question to elucidate the underlying mechanisms of host-microbiome interactions considering the high-integration property of OOCs. Initially, 3D HOs, microbiome and immune cells can be isolated from healthy persons or patients to realistically refactor essential factors of the intestinal ecosystem architecture. In the future, establishing human 3D organotypic models derived from healthy persons or patients are expected to be used for practical application. Altogether, the OrgOC methodology could be employed to illustrate the complex interactions between host cells and the microbiome within the human body (e.g. lung, skin, urogenital, etc), thereby broadening our understanding of human diseases and advancing drug development.

#### 3.1.4 Inter-organ communication

In the body, most physiological pathways require continuous media circulation and physiological communication of the organs. However, current OOC models usually have a single organ that possessed the oversimplified niche and cannot fully reflect the complexity, functionality and integrity of *in vivo* organs due to the lack of physiological interactions between organs. In order to simulate the complex human organs, establishing a more complex multi-organ-chip system is needed. *In vitro*, researchers have attempted to combine different organ equivalents into a human-like ecological environment in a dynamic micro-reactor. Multi-organ-chip provides a new mode to culture different organs in separate spaces connected by microfluidic channels, which incorporates a micropump to ensure circularly dynamic conditions, in which each tissue/organ is cultured in its specific microenvironment and linked by continuous flows.

At present, there is no accepted standard of how to equate human organs *in vitro* with real human body, which to a large extent leads to the design of chips being unfounded. Energy metabolism represents the most important biological function, as organisms depend on energy to carry out bodily behaviors. Based on this tenet, Marshall's team presented and tested a new theory, allometries of metazoan life, that predicts the interactions among three fundamental aspects of life (metabolic rate, growth, and reproduction) 233. Guzzardi et al. adopted allometric scaling methods to develop a simplified downscaled human body *in vitro* in a multi-compartmental chip based on the crosstalk between hosting livers and endothelium 234. However, due to the use of extra pumps and bulk reservoirs, the organ volume is a very small fraction of the total system, and signaling molecules are significantly diluted. To solve this problem, Marx's group presented another scaling law, that is setting the scaled organ compartments and related parameters (size, composition, and flows) of organ chips according to the ratio of the tissue size *in vivo* 235. Wagner et al. designed a liver-skin-chip, each a 1/100 000 of the biomass of their native human organ equivalents, in which organ chambers were shielded from the bottomed microfluidics by standard transwells 235. Unlike the previous reports, this work introduced a peristaltic micropump and an infinitesimal media setup into a chip, minimizing the fluid-to-organ ratio within the whole system and ensuring the tunable velocities of the media flow. Although these two scaling modes of human organs on a chip are different, we can gradually grow closer to the truth through scientific research and it is undeniable that the function of organs can be enhanced in a co-culture system than that in a mono-culture chip owing to the unique advantages of OOCs technology.

As mentioned in the above section, HOs can better reflect the functional properties of human organs, so the researchers have shifted their focus to the construction of multi-organoid chips. Qin's team recapitulated the human liver-islet axis based on organoids derived from hiPSCs in a circulatory perfusion system 10. Notably, the co-culture system was found to promote insulin secretion in response to glucose and enhance glucose uptake in the liver. Furthermore, the glucose levels decreased and returned to normal fasting levels rapidly in the liver-islet axis model, which was not found in the mono-organoid culture. Under high glucose treatment, the liver-islet axis displayed impaired mitochondrial function and glucose transport, which can be alleviated by metformin. These results showed the capacity of the OOC system in modeling physiological postprandial blood glucose metabolism and hyperglycemia in type 2 diabetes mellitus (Figure 4B) 10. Yin et al. designed a sandwiched chip coupled hepatic and cardiac organoids separated by a porous membrane 90. In this model, high viability and organ functions were maintained in two kinds of organoids. In particular, hepatic organoids displayed much stronger urea synthesis and metabolism-related CYP450 enzyme activity in the co-cultured system. The human body is a complex organic combination and every organ works with each other linked by blood circulation. To reflect the whole human body, three or more organs/organoids were implemented based on OOC systems 163, 164, 213. To more accurately demonstrate inter-organ physiology and biochemistry at a multi-organ level, Jin et al. constructed an integrated liver, stomach, and intestinal organoid model in a high-throughput microfluidic setup, allowing to explore drug metabolism and the bile acid-induced regulation 162. The report demonstrated a valid crosstalk between these organoids likely due to the secretion of autocrine and paracrine signals from organoids by a well-defined engineering strategy (Figure 4C).

In academia, one of the main scientific concerns is how to develop a universal blood substitute medium to ensure the functionality of all organs in the same recycle system. Ingber group has developed a common blood substitute medium, which can maintain the viability and organ-specific functions of eight organs-on-chips 236. In this microsystem, the organs including the intestine, liver, kidney, heart, lung, skin, blood-brain barrier, and brain can be well maintained for up to 3 weeks. Multiple organs were linked by vessel-like channels, which were patterned with human parenchymal cells and vascular endothelium. Moreover, the automated culture setup was established by the combination of liquid-handling robotics, a custom software package, and an integrated mobile microscope. This intelligent system allows for medium addition, perfusion, cell imaging and sample collection of up to microdevices inside a standard cell culture incubator 236. Obviously, these devices have displayed the advantages of finely adjusting the rate and volume of dynamic flow during long-term culture. Up to now, only the fluid aspect is considered in a biomimetic multi-organoid-on-a-chip. In the future, aiming at individual organs with unique properties, more complex organ-specific micro-environmental factors (e.g., mechanical force, oxygen/concentration gradient, and matrix hardness) should be incorporated to better simulate the human system.

### 3.2 Disease modeling

Recent progress in HOs, gene editing and directed differentiation technologies has provided opportunities to develop state-of-the-art human disease models 18, 19. As promising artificial living systems, OrgOCs enable researchers to witness and study various pathologic aspects of the organism in an unprecedented way, including disease occurrence, pollutants exposure, and viral infection. In this section, we will follow up on the latest developments in the field, including endogenous component causes, pollutant exposure and viral infection.

#### 3.2.1 Endogenous component causes

Up to now, many disease-on-a-chip models have been developed, such as cystic fibrosis (CF), liver diseases, tropical enteropathy, inflammation, thrombosis, Parkinson's disease and tumor metastasis 34, 73, 122, 129, 237, 238. These disease models can undoubtedly uncover novel mechanisms, specific targets of disease pathogenesis and their epigenetic regulation mechanisms. For instance, various human organoids have been utilized to investigate metabolic diseases, infectious diseases, inheritable genetic disorders and cancers 26, 31, 106, 239, 240. Notably, with the advent of various genetic engineering tools, such as CRISPR/cas, transposase and RNAi, isogenic disease models with healthy donor-derived organoids can be constructed, enabling to elucidation of pathological properties and even tissue repair. To construct a disease model, cells can be either obtained from patients with particular diseases, by inducing genetic mutation in healthy cells, or even by adding the pathogenic factors during the development of healthy cells.

**Figure 5:**
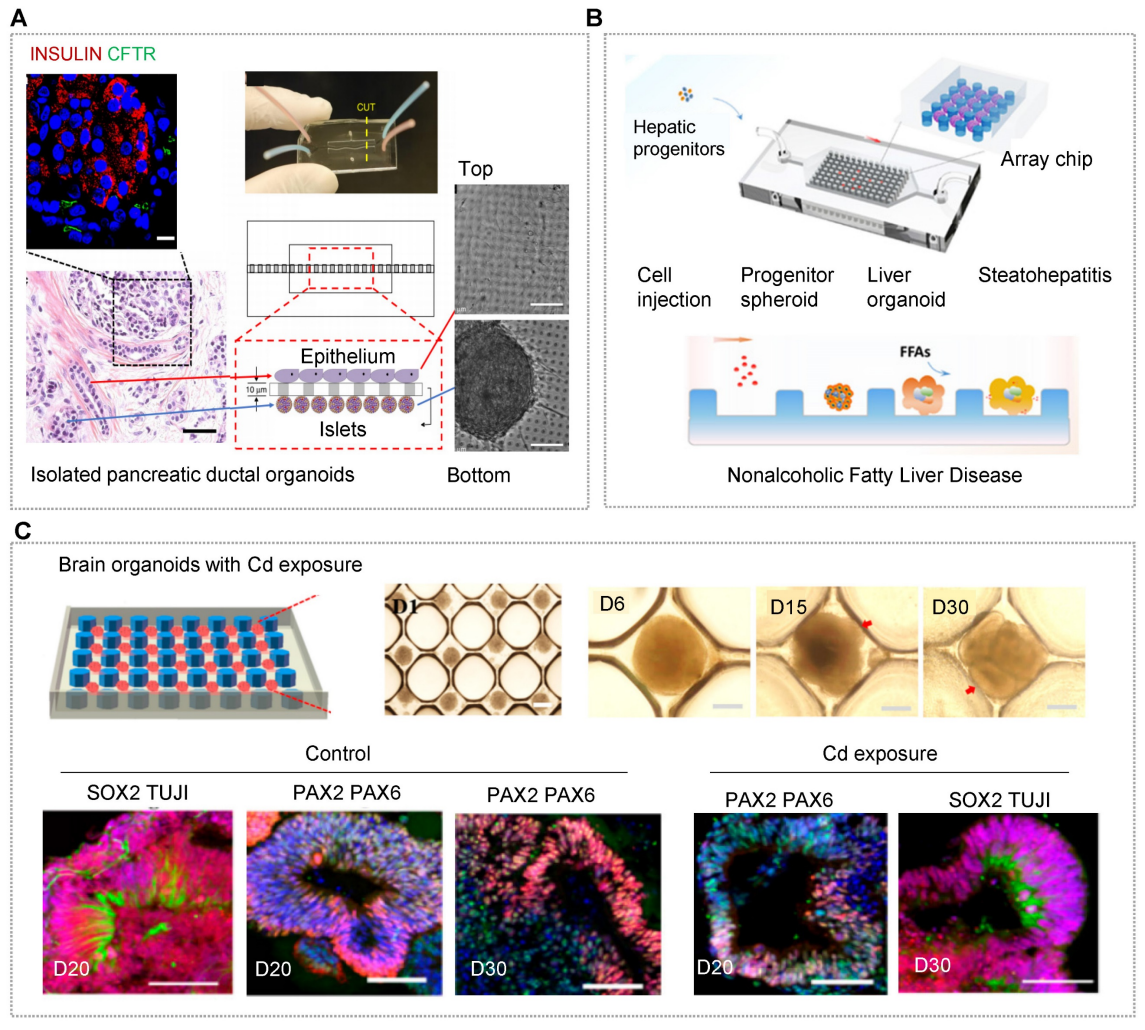
OrgOCs platform for disease modeling. (A) Patient-derived pancreatic ductal organoids-on-a-chip can recapitulate cystic fibrosis-related disorders and examine the cell-cell functional correlation between pancreatic ductal epithelial cells and islets. Adapted with permission 250. Copyright 2019 Springer Nature. (B) With free fatty acids, liver organoids from hiPSCs exhibited the typical pathological characteristics related to steatohepatitis. Adapted with permission 34. Copyright 2020 American Chemical Society. (C) Heavy metal cadmium exposure might cause precocious, lasting neural differentiation and long-term neurotoxicity in brain organoid development with a chip. Adapted with permission 132. Copyright 2018 American Chemical Society.

Notably, patient-derived OrgOCs offer unprecedented opportunities to develop human pathological models with identical genetic information and high fidelity. Patient samples from colon, brain, prostate, pancreas, liver, breast, bladder, stomach, oesophageal, endometrial and lung cancers have already been used as disease models 95, 130, 155, 241-247. It is worth mentioning that cancer organoids from cancer resections and metastasis biopsies recapitulate tumor histopathology and gene mutation status, allowing for xenotransplantations and drug screening 27, 248-251. In addition, many genetic disorder diseases can also be recreated by organoids, such as cystic fibrosis (CF) featured with the defective transmembrane conductance regulator (CFTR) function. Naren and his partner modeled CF-related disorders using a patient-derived pancreatic ductal organoids-on-a-chip co-cultured with islet cells (Figure 5A) 252. In the study, authors examined functional correlations between pancreatic ductal epithelial cells (PDECs) and islet cells. Surprisingly, the inhibition of CFTR function in PDECs was found to reduce the secretion of insulin, suggesting the functional coupling of ductal cells and islets. Ingber's group has recreated many features of the living human intestine by culturing patient-derived organoids on physiological gut chips 253. Based on these works, his group made a breakthrough in simulating pathological states of the intestine, such as nutritional disease and inflammatory bowel disease. Typically, Bein et al. remodeled tropical enteropathy by culturing organoid-derived intestinal epithelium from patients with environmental enteric dysfunction (EED) in a nutrient-deficient medium without niacinamide and tryptophan on a classic sandwiched chip 154. The *in vitro* model recapitulates the transcriptional signatures of EED patients and severe EED‐associated intestinal injury features, including villus atrophy, barrier dysfunction, reduced amino acid uptake and metabolism, and inflammation. Thus, this gut-on-chip model enables clinicians to explore multiple clinically relevant outcomes and distinguish the manifestations of multiple intestinal diseases from EED.

Human stem cells-derived organoids are generated through the sequential addition of different growth factors following the intrinsic developmental programs. According to specific situations, several pathological models were established by adding exogenous reagents. For example, nonalcoholic fatty liver disease is one of the main causes of chronic liver diseases worldwide, and is directly associated with obesity, insulin resistance, and hypertension. Wang et al. presented a nonalcoholic fatty liver disease (NAFLD) model based on a perfused OrgOC system 34. Under exposure to physiologically relevant free fatty acids, NAFLD organoids exhibited the typical pathological characteristics of steatohepatitis, including abnormal lipid metabolism, triglyceride accumulation, oxidative stress, inflammatory response, and fibrosis (Figure 5B) 34. Another report established a disease model of diabetic nephropathy, one of the most serious chronic microvascular complications. In this study, Wang et al. assessed the changes in the glomerular barrier in phenotypic expression, reactive oxidative stress and barrier integrity under pathological conditions 254. In addition to common metabolic and progressive diseases, hiPSC-derived organoids as an ideal cell source have been exploited to model infectious, genetic and nutritional diseases and so on 255. These models can be used to assess short-term or long-term effects of drugs/stimuli on specific tissues/organs. All in all, these works demonstrate the potential of OrgOC as a useful platform to accelerate our understanding of pathological mechanisms.

#### 3.2.2 Inorganic pollutant exposure

The atmospheric pollutants exposure poses health risks and a significant socioeconomic burden. It is a major concern of the new civilized world that deserves high attention to explore the underlying pathogenicity of atmospheric pollutants on human health, such as chemicals, inorganic nanoparticles (NPs) and particulate matters (PMs). OrgOCs provide new opportunities to comprehensively understand the mechanisms of atmospheric pollutants and monitor the biological effects of the substances t pollutants exposure on human organs.

The researchers have utilized OrgOCs models to explore the adverse influences of chemical pollutants on human organs 122, 132, 147, 255. It is worth discussing that prenatal exposure to environmental pollutants can raise the risk of developmental diseases. Qin's team created many OrgOC models to investigate neural dysfunctions of human brain organoids by environmental factors exposure including heavy metal cadmium (Cd), nicotine, alcohol and valproic acid (VPA) 122, 132, 147, 255. With Cd treatment, distinct cell death was observed in brain organoids, even 10 days after the removal of Cd, indicating the long-term neurotoxicity of Cd on brain development 132. The findings showed that Cd exposure might cause precocious and lasting neural impairments in neurogenesis and brain regionalization during early development (Figure 5C). In addition to the chemicals mentioned above, cigarette smoke is another risk of clinical exacerbation in patients with chronic obstructive pulmonary disease (COPD), that cannot be effectively imitated in animal models. Scientists have developed a programmable microfluidic system that enables to dynamically mimic the whole process of smoking and model smoke-induced COPD exacerbations 256. This approach offers an analytical tool to illustrate the reduced mucociliary clearance and ciliary dysfunction observed in the lungs of smokers, as well as the development of squamous metaplasia observed late in the pathogenesis of COPD. In the organotypic culture model, the key marker MMP-1 was found to be involved in the COPD pathogenesis response, and a similar phenotype (oxidant damage) can be observed in COPD patients and smokers 256. Thus, these systems have the great potential to identify subtle yet potentially clinically relevant changes in gene expression, that might otherwise be neglected in studies involving individuals or other heterogeneous patient populations. Overall, the organ chip is not meant to directly extrapolate the findings to the clinic, but it is easier to discover specific biomarkers and therapeutic targets than other existing technologies.

Inorganic nanoparticle (NP) exposure has raised issues about their potential toxicity due to their extensive use in our daily lives 36, 237. In recent years, more and more attention has been paid to the effects of NPs, especially ultrafine NPs (aerodynamic diameters < 100 nm), on human health. Ingber's team has found that NP-induced ROS production was proportional to the level of physiological breathing strain applied 36. Breathing strain could dramatically induce the proinflammatory activities of NP, and might lead to the onset of acute lung inflammation. Importantly, NP transport across the alveolar-capillary barrier was up to 4 times higher under physiological breathing motions (10% strain at 0.2 Hz) than static culture. Strain induced the increased NP absorption via the impaired transcellular translocation or paracellular transport, rather than physical disruption of cell-cell junctions and convective transport 36. The model contributes to analyzing the mechanisms underlying biological phenomena, which can't be done with traditional models.

#### 3.2.3 Viral infection

Both clinical and animal findings showed that viral infection can cause a variety of diseases 257-261. For example, human immunodeficiency virus (HIV) infection can result in long-term neuropsychiatric adverse effects (neurotoxicity, depression, anxiety, and mood disorders) on individuals 26, 83, 260, 262. Due to species differences in animal and human biology, it is highly desirable to develop a robust, available and humanized organ model that reflects human pathological features with viral infection. Organ chips present a rapid and effective tool to explore viral infection and transmission dynamics in a realistic manner 257, 260, 263, 264. Morphological analysis of respiratory syncytial virus-infected airway organoids uncovered a wide range of impairments ranging from cytoskeletal rearrangements and apical extrusion of infected cells, to syncytia formation 265. Zika virus (ZIKV) infections could lead to severe congenital abnormalities, as evidenced by the stunted cortical expansion and microcephaly in human cerebral organoids 230.

Unlike common infectious diseases, severe acute respiratory syndrome coronavirus 2 (SARS-CoV-2) is mutational, contagious and deadly, leading to acute respiratory distress, other complications, or even death 266-270. Facing this pandemic, various tissue barriers were designed to model the viral infection. Zhang et al. reproduce the SARS-CoV-2 infected disease model using a human alveolar chip with lung epithelium, endothelium and immune cells 263. In this work, viral infection resulted in alveolar barrier injury, immune cell recruitment, exacerbated inflammation, and higher susceptibility to the virus was identified in epithelium compared to the endothelium. Additionally, Kuo and his coworkers created distal lung organoids derived from human alveolar epithelial type II (AT2) or KRT5^+^ basal cells 271. With SARS-CoV-2 infection, about 10% of AT2 and bassal organoids displayed prominent SARS-CoV-2 nucleocapsid protein (NP) expression. However, NP- or dsRNA-positive signals mainly existed in club cells, accounting for 79% of NP- or dsRNA-positive cells in all infected cells. Overall, these studies implicate club cells as a target of infection in addition to AT2 cells.

Clinical evidence validates that the intestine is another target organ for SARS-CoV-2 infection. Based on the OOCs model, Qin's group further explored the effect of SARS-CoV-2 on human gastrointestinal injury 272. Under the virus infection, the intestinal barrier showed significant destruction, including morphological abnormity of villi, sparse mucus secretion, upregulated cytokine genes and abnormal metabolism. Collectively, these models can closely mirror human-relevant responses to SARS-CoV-2 infection, overcome the shortcomings of traditional models, and provide a promising strategy to accelerate COVID-19 research and drug development. Together, organ chips can propose a versatile platform for the full investigation of viral transmission and evolution between humans by passaging viruses from one chip to another, and thereby facilitate the identification of possible intervention strategies to cope with emerging variants.

### 3.3 Precision medicine

#### 3.3.1 Pharmacokinetic study

Generally speaking, the birth of a new drug needs to go through a series of processes, such as the selection of drug targets, the determination of lead compounds, the selection of drug candidates, the evaluation of safety and efficacy in animals, and the clinical trials in humans 273-275. As animal models are still far from the human body, there is a great need for biomimetic disease models to reduce the cost and the time of new drug development. As an emerging field, organ chips aim to make organoids easier to operate and control, so as to reflect the complex internal environment of the human body as comprehensively as possible. Organoid chips have the characteristics of miniature structure, high throughput and sensitivity, and integration of a series of experimental processes, such as organoid culture, observation, selection, induction, detection and analysis into one system 276. One area of organoid study that is ripe for further development is quantitative pharmacokinetic/pharmacodynamic (PK/PD) modeling. With the precise control of relevant parameters, OrgOCs can function as *in vitro* human organ microsystems with enhanced physiological maturation and function compared to cell monolayers, and better predict human outcomes of multiple perturbations compared to animal models 16, 17, 140. Hence, OrgOCs are promising to narrow the gap between animal studies and clinical trials, potentially promoting drug development. Of particular note, living biobanks of cancer organoids contain massive samples with different genetic backgrounds, subtypes and individualized properties, and offer a powerful platform for drug screening and discovery 243, 277, 278. Moreover, the development of OrgOC test platforms with controlled cancer organoids and near-physiological microenvironment will further promote the predictive ability. Even so, how to correlate data from organoids with other bioengineered systems remains a prominent hurdle.

As a new iterative model, organ chips have great potential in the multi-trillion track of pharmaceutical research and development 82, 160, 275. It is expected to bring changes to the drug development industry by shortening the discovery cycle, reducing costs and improving the success rate of drug candidates. In early 2021, the Food and Drug Administration (FDA) released a white paper expressing its positive attitude toward the application of organ chips in the development of novel drugs, and describing the potential of standardized OOC systems to replace the animals as the preferred models. With the support of domestic policies, pharmaceutical companies also begin to pay attention to and be optimistic about the practical application value of organ chips. Now, more and more pharmaceutical companies advocated for the use of organ chips in biomedical research, and a breakthrough is expected in the next 3-5 years.

From entering the body to leaving the body, drugs undergo absorption, distribution, metabolism and excretion, that is, pharmacokinetics (PK), which is related to the dose of the drug and determines the time course of drug concentration in serum, body fluids, and tissues. On the other hand, the mechanism of action and the relationship between drug dose, efficacy, toxicity should also be studied, that is pharmacodynamics (PD). In the body, drugs go through a series of complex physical, chemical and biological changes. Some substances that entered the body through diet do not exhibit biological activity until hydrolyzed in certain organs by specific enzymes to remove certain groups from the molecule. Accumulation of active metabolites increases the risk of organ damage (e.g., liver function impairment, myocardial toxicity, neurological disorders, and abnormal brain development) 14, 279, 280. Thus, it is promising to establish multi-organ models to overcome the lack of suitable *in vitro* models for drug development, which has attracted great attention from pharmaceutical enterprises.

The liver is the main organ of drug metabolism and thereby largely determines the pharmacological properties of drugs, including bioavailability and adverse effects. Lin's team from Tsinghua University utilized a double-layered multi-organ chip to recapitulate the intestine-liver functionality for studies in the absorption, transport and metabolism of combination drugs (genistein and dacarbazine) 281. The results suggested that there is no obvious cell cycle arrest of liver cells under the low dose (<100 μg/mL) treatment of drugs, however, severe cell apoptosis could occur at levels above 200 μg/mL, verifying the effective drug metabolism on a chip. In the liver-heart axis model, liver organoids displayed higher liver metabolic capabilities and hepatic metabolism-dependent cardiotoxicity 90.

**Figure 6:**
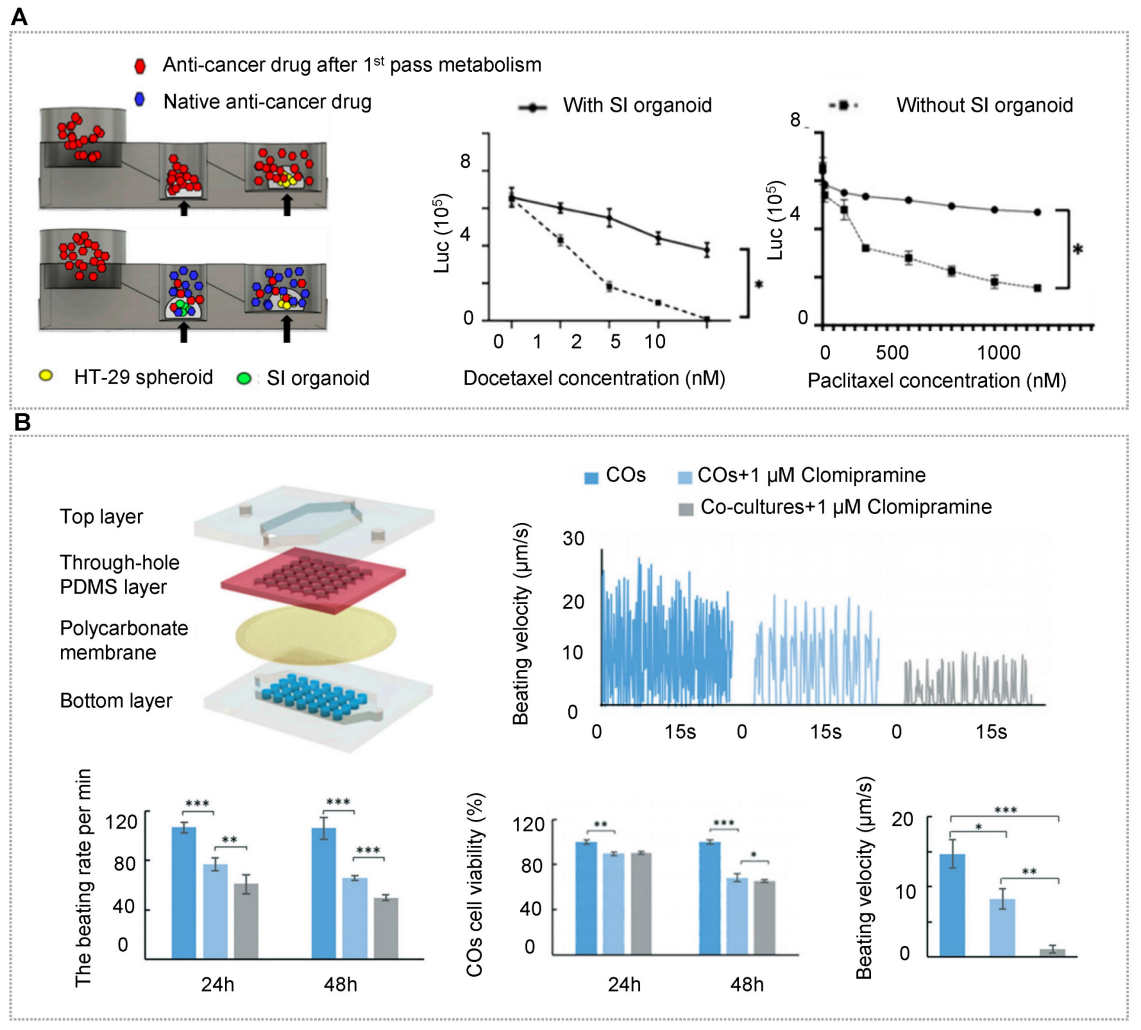
OrgOCs platform for assessing drug metabolism and safety in precision medicine. (A) Recapitulation of first-pass metabolism of anti-cancer drug with intestinal organoids-on-chips. Adapted with permission 281. Copyright 2021 Multidisciplinary Digital Publishing Institute (MDPI). (B) Liver-heart organoids-on-chips allowed to explore the hepatic metabolism-dependent cardiotoxicity of clomipramine. Adapted with permission 284. Copyright 2021 AIP Publishing.

Systematic interactions at the multi-organ level are considered to be crucial to determining drug metabolism and the pharmacological effects of various drugs. Thus, it is expected to establish multi-organ-chips systems with the characteristics of systematic interactions, possibly reflecting the complex drug metabolic processes and actual drug responses. A major obstacle in drug development is the low bioavailability of oral drugs due to first-pass metabolism 282. To cope with the challenge, developing *in vitro* OOCs systems have been applied for pharmacokinetic study. Ingber's group has constructed a first-pass metabolism model with the gut, liver and kidney linked by endothelium-lined vascular channels on a chip 282. This model could reflect the key clinical pharmacokinetic parameters including drug absorbance, metabolism and clearance with oral administration of nicotine and intravenous cisplatin. It is worth mentioning that the obtained PK/PD profiles were in accordance with clinical studies, suggestive of the enormous potential to predict pharmaceutical responses of drug candidates. In another work, Lee et al. analyzed the effect of first-pass metabolism using docetaxel 283. In the chip without small intestinal organoids, the viability of colorectal adenocarcinoma spheroids was significantly reduced. However, in the chip with small intestinal organoids, no significant change in the viability of colorectal adenocarcinoma was observed due to first-pass metabolism (Figure 6A) 283.

#### 3.3.2 Drug safety assessment

There are great challenges to medication safety because it is hard to balance the health of patients with the potential adverse effects of drugs. Drug development is a lengthy and costly process, partially owing to the use of preclinical models with low accuracy of efficacy and toxicity prediction of drug candidates. The cutting-edge organoid chips are leading a revolution in the safety testing of compound candidates, providing an appropriate therapy selection in the premarketing phase.

In order to more precisely reflect drug safety at the physiological level, researchers are shifting from OOC to OrgOC technology with multiple organs that can systematically respond to stimuli. Yin et el. assessed drug-induced cardiotoxicity with liver-heart organoids-on-a-chip 90. With exposure to clomipramine (a tricyclic antidepressant) in liver organoid chamber, heart organoids in the bottom chamber were found to display impairments in cell viability, cardiac beating and calcium activities, suggesting the hepatic metabolism-dependent cardiotoxicity of clomipramine (Figure 6B) 284. This impressive multi-organoids-on-a-chip system can recapitulate the complex process of drug metabolism at the multi-organ level, and be expected to function as a powerful tool to evaluate drug candidates. Skardal and coworkers probed prodrug metabolism and reciprocal toxicity in a three-organoids-on-a-chip system 163. Prodrug capecitabine (a chemotherapy drug) can translate into cytotoxic 5-fluorouracil (5-FU) in the liver chamber and then act on lung and cardiac organoids. Compared to the system without the liver, cell viability of the heart and lung decreased 38.3% and 37.1% respectively after liver metabolism 163. In the expanded 6-organoids system, similar results showed that chloroacetaldehyde, produced by the metabolism of the alkylated prodrug ifosfamide in liver organoids, can induce significant neurotoxicity.

In addition to chemical drugs, organ chips can also be employed to screen biomarkers of toxicity. Kacey et al. proposed a OOC model with human heart, liver, bone and skin organ niches, which were linked by the recirculating vascular flow 41. With *in vivo*-like pharmacokinetic and pharmacodynamic profiles of doxorubicin, this systematic model enabled to identification of early miRNA biomarkers of cardiotoxicity. In the future, it will be possible to systematically assess the toxicology of more than one drug at the same time in a high throughput manner. It's worth noting that drug bioavailability determines the amount of circulating drug in the body that remains active for an established period. However, one major drawback of PDMS is its hydrophobic surface and high porosity, which potentially results in the absorption of many hydrophobic molecules. An alternative can be developed such as polycarbonate, glass, polystyrene, and poly(methyl methacrylate) (PMMA), which are known to be inert to most drugs, contributing to the increased accuracy of drug evaluation 6. Besides, more accurate empirical methods, such as mass spectrometry, can further promote quantitative accuracy for drug bioavailability. However, it will be challenging to integrate these techniques with the OrgOC system given the small fluid volumes involved.

#### 3.3.3 Drug screening

**Figure 7:**
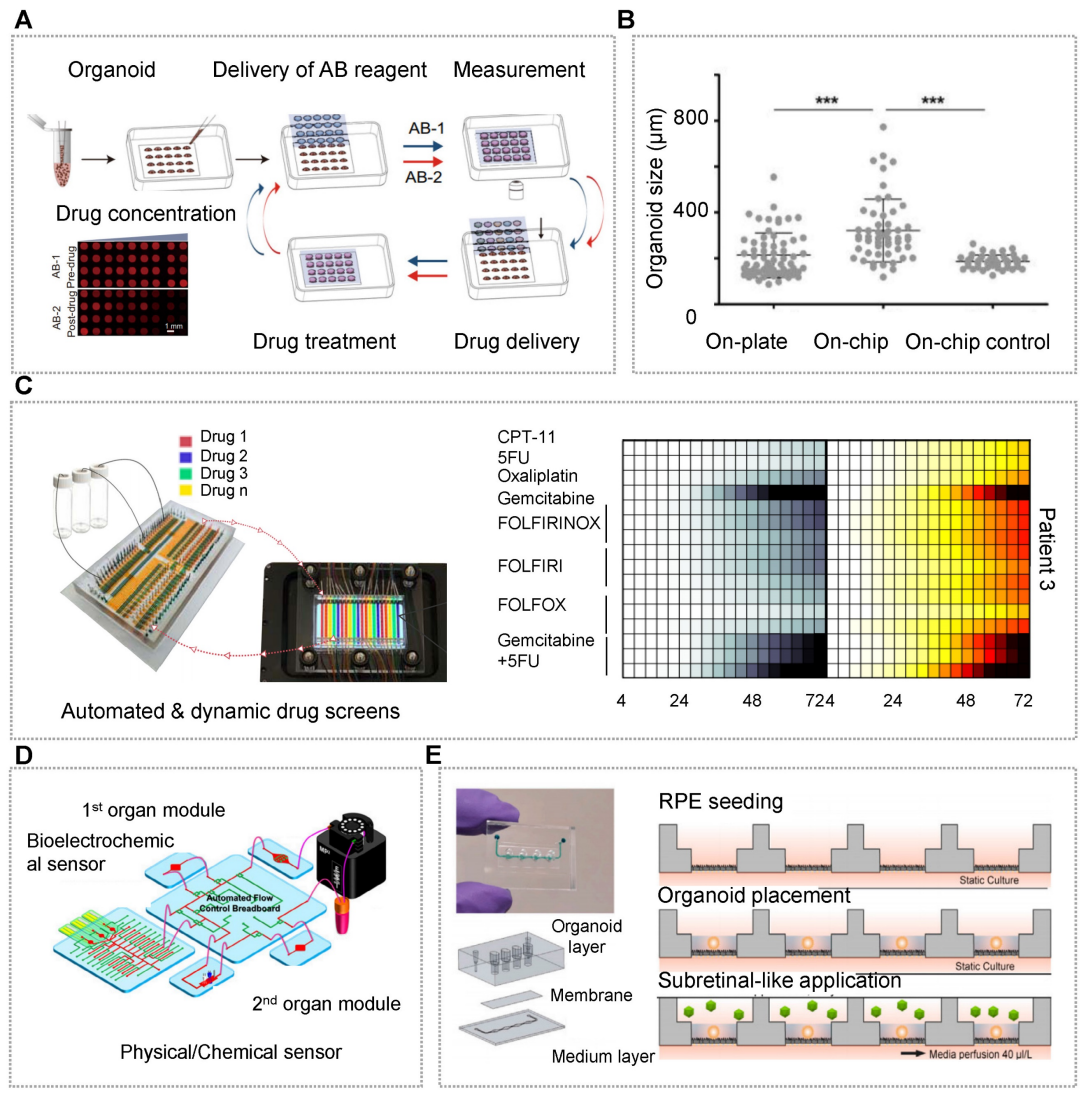
OrgOCs platform for drug screening in precision medicine. (A) Hundreds of lung cancer organoids produced from clinical specimens were generated at passage 0 by coupling with a microarray chip device. Adapted with permission 131. Copyright 2021 Springer Nature. (B) A nested array chip platform cultured patient-derived colorectal cancer organoids for high-throughput drug screening. Adapted with permission 150. Copyright 2021 MDPI. (C) A one-stop microfluidic-based lung cancer organoid culture device for drug sensitivity test. Adapted with permission 160. Copyright 2020 Springer Nature. (D) An organoid chip system with an automation controller coupled with modular multisensor (e.g., physical, biochemical, and optical sensors) units via a fluidics-linked breadboard for automated evaluation of the therapeutic responses of organoids to drugs over extended periods of time. Adapted with permission 161. Copyright 2017 PNAS National Academy of Sciences. (E) A human hiPSC-based retinal organoids-on-chip model was employed to test the transduction efficacy of gene therapy with different types of AAV vectors in a pharmaceutical setting. Adapted with permission 156. Copyright 2021 ISSCR.

High-throughput screening (HTS) is a practical approach that enables automated testing of massive potential drug candidates against a biological target and is extensively used in the pharmaceutical industry. Thus, the building of a living organoid biobank will dramatically advance drug screening. Living organoid biobank contain various organoids for personalized therapy and research in life science. Especially, as a biomedical resource, the establishment of tumor organoid biobanks contributes to preclinical models, drug screening and personalized therapy. Traditional* in vitro* models are frustrated to preserve the cellular fidelity and mutant multiformity of tumors and are usually time-costing 285, 286. Currently, more and more scientific research institutions and enterprises have built patient-derived cancer organoid biobanks to capture disease heterogeneity and genotype-phenotype mapping 154, 277, 278. For instance, Song's team exploited a novel protocol to produce patient-derived glioblastoma organoids, retaining key hallmarks of glioblastomas (e.g., original cell-cell interplay, inter-/intra-tumoral heterogeneity, and mutational profiles of native tumors), and avoiding the destruction of local tissue usually occurred using traditional methods (e.g., mechanical or enzymatic dissociation) 277. Besides, it highlights the potential of cancer organoids in investigating patient-specific therapies by correlating cancer organoid mutational profiles with responses to specific drugs and modeling chimeric antigen receptor T cell immunotherapy. We believe more live biobanks will be established for basic research and clinical transformation. Notably, living organoid biobanks, combined with drug sensitivity testing and next-generation sequencing, now support clinical decision-making and clinical trial performance analysis. Liu and coworkers efficiently generated hundreds of lung cancer organoids produced from clinical specimens at passage 0 by coupling with a microarray chip device (Figure 7A) 131. It is worth mentioning that the one-week drug sensitivity testing results were well consistent with patient-derived xenografts, tumor gene mutations, and clinical findings. To date, more and more organoids-on-chips, including lung organoids-on-chips, intestine organoids-on-chips, liver organoids-on-chips, vessel organoids-on-chips, pancreas organoids-on-chips and so on, have been applied in monitoring organoid behavior *in vitro*, drug screening and drug development 123, 127, 149, 157, 160, 287.

Ai's group exploited a nested array chip platform cultured patient-derived colorectal cancer organoid for HTS 123. In the report, the authors used nine treatment regimens to test the practicability of the platform and detected the growth kinetics of the organoids in different culture modes. Notably, the patients with rectal cancer and liver metastases showed more sensitivity to regorafenib but not to fruquintinib, suggesting the anti-tumor and anti-angiogenesis activity of regorafenib. Meanwhile, the drug sensitivity testing based on the OrgOCs models showed good quality control with a coefficient of variation under 10% 123. The advanced OrgOC microdevice overcomes the limitations of the conventional plate well-based platform and enables the organoids to grow in a precise manner (Figure 7B) 150. Also, OrgOC microsystems demonstrated that the core of organoids was chemo-resistant, indicating the different drug sensitivities in different parts of organoids 149.

Automated microfluidic chips and artificial intelligence technology have unique superiority of high-throughput detection and precision analysis of cells matched with organoids. Unlike traditional plate-based methods, automated microfluidic chips allow for combination therapy with sequential pulse delivery to the desired array of organoids without human intervention, requiring no manual operations, thereby reducing human errors while enabling dynamic imaging. The novel platform can not only verify standard medical therapy practices, but also optimize the program to help the pre-clinical program in an economical and reliable way (Figure 7C) 160. Khademhosseini's team developed an organoid chip system with an automation controller coupled with modular multisensor (e.g., physical, biochemical, and optical sensors) units via a fluidics-linked breadboard, which can achieve an accurate, continual, and automated evaluation of the therapeutic responses of organoids to drugs over extended periods (Figure 7D) 161. Besides, multiomic analysis contributes to revealing cancer-related noncoding mutations, gene regulatory routing and chromatin signatures associated with drug sensitivity, offering new insights into cancer occurrence and drug treatment 287. Overall, these breakthrough studies demonstrate that the OrgOC platform can offer opportunities to monitor tumor progression, assess therapeutic modalities and understand chemotherapy resistance. We can believe that the OrgOC platform will provide compelling opportunities to promote precision medicine for cancer at the preclinical level.

OrgOC platform can also be used to evaluate the efficacy of gene therapy. For instance, Loskill's group employed a hiPSC-based retinal organoids-on-chip model to test the transduction efficacy of gene therapy with different types of adeno-associated viruses (AAV) vectors (scAAV2, scAAV9, scAAV2.7m8, and scShH10) (Figure 7E) 156.

## 4. Conclusions and Perspectives

The organisms are regulated by many physicochemical and physiological cues, which can be utilized to create synthetic living systems. Therefore, the emergence of OrgOCs represents a major breakthrough and starts a new page toward the construction of bio-artificial organs by remodeling organotypic multicellular architectures and functions in a defined ecological niche. At present, most human organoids have been recreated on microfluidic chips, including the intestine, brain, kidney, liver, and islet, with near-physiological properties of *in vivo* organs 16, 150, 288. To achieve systematic interaction, long-term and stable coculture of multiple organs, programmable dynamic flow is applied to the microfluidic system that connects multiorgan modules to accurately mimic blood circulation* in vivo*. Together, this systemic communication or mutual feedback is vital to understanding and simulating several temporal physiological processes. Multiple pathological processes can be well reflected with OrgOCs platforms, including disease occurrence, pollutant exposure and virus infection. Development of organoid chips that reproduce complex physiological and pathological responses to external and internal stimuli at the organ level could revolutionize biomedical fields, involving biological study, disease modeling, preclinical assay and precision medicine that rely heavily on animal experiments and clinical studies. The general principles involved in the development of organs and organ physiology derived from this approach have resulted in a deep understanding of many human disorders. Ideally, organ chips can recapitulate the *in vivo*-like PK/PD profiles and quantitatively predict human pharmacokinetic responses to drugs, involving absorption, distribution, metabolism, excretion, toxicity in a specific order. OrgOC models can also be applied for drug screening and sensitivity detection in a high-throughput and high-bionic trait manner, which is not possible in traditional cell and animal models. A key point in modeling complex and heterogeneous disorders is understanding inter-individual differences. Uniquely, patient-derived personalized organoid chips can model patients' comorbidities and give specific drug selection, including optimized drug efficacy, minimized toxicity, and even optimal delivery routes, as well as optimal dosing regimens for use in targeted phase I clinical trials. Moreover, engineering strategies allow us to efficiently obtain organ models with complex morphological properties, geometric features and inbuilt quality attributes, similar to those of native tissues. Organoids derived from stem cells as a scalable cell source have the potential to promote wound healing and tissue regeneration. Cell therapy and gene therapy also reflect the practical application value of the OrgOC platforms, which can accelerate the progress of disease therapy. Therefore, OrgOC is expected to speed up the preclinical-to-clinical translation in medical and industrial fields.

Indeed, many pharmaceutical and academic researchers are wary of changing the ways they carry out research. Or say, convincing data to demonstrate the advantages of human organ chips over animal models are needed, before they accept this new technology. That is one reason that, despite the great promise of OrgOCs, researchers have not formulated a universally accepted gold standard, leading to the difficulties of preclinical-to-clinical translation. To address the existing limitations, efforts need to be made to achieve more biomimetic human tissue models. The human body is a complex organic system, with various organs interacting and influencing each other, among which the vascular system, responsible for blood circulation and material transport in all parts of the body, is an important part. Therefore, it is important to maintain organoid chips in a perfusable mode. At present, a common strategy used is to improve the vascularization and maturation of organoids by guiding the self-assembly of endothelial cells 289. In addition, *in vitro*-like environmental features such as ECM and dynamic flow have been considered to optimize the culture system of vascularized organoids 289. In addition, to mimic the immune response, it is essential to incorporate immune cells into the organoids. Infiltration of monocyte into vascularized organoids was successfully reconstituted on a chip 137. Despite great efforts that have been made to form functional vascularized or immune cell integrated organoids, the formation of fully vascularized or immune organoids is still a major challenge so far. Future work will seek to develop more complex organoid co-culture systems incorporating multicellular types and more types of immune cells. It is worth thinking about to what extent the organoids can replace real human organs or reflect the physiological responses. So, on the one hand, the criteria for optimizing OrgOCs should consider environmental parameters, organoid size, chip design, etc. On the other hand, researchers exploiting OrgOCs platforms ought to standardize and validate these models against human samples and clinical data as accurately and feasible as possible. Nonetheless, there are now standards and criteria to identify the use of human organ chips to replace animal models, and important advances are being seen in this field. We are now facing a tipping point in this era, with the possibility of seeing real reductions in the use of animals and the application of more effective approaches for drug development, personalized medicine as well as basic research in the near future. The aim of next-generation OrgOCs is to systematically reconstruct an actual and miniature human body *in vitro* with universal and extendable advantages.

The solo model has a limited ability to meet the requirements needed to address biological and medical applications, especially for emerging and re-emerging pandemic diseases. The newly developing OrgOCs technology has exhibited great potential in building organ models with higher fidelity and practicability. 3D bioprinting technology and biological materials can also be incorporated into the OrgOCs platforms to increase the complexity of organs in structure and function, expediently constructing original human organs or tissues with biomechanical characteristics similar to those *in vivo*. In addition, microscope imaging technology can meet the requirements of real-time tracking, analysis of human organ development, lesions, treatment progress, and monitoring of human diseases, allowing real-time visualization of the whole dynamic process 148, 290. Multi-omics can analyze the pathology and disease state of organs *in vivo* at the molecular levels, which is helpful for the discovery of disease markers, signal pathways and potential therapeutic targets of drugs. The burgeoning techniques are expected to be the standardized strategies for uncovering biological phenomena, including homeostasis and physiologic disorders within and beyond human organs. We assume that by incorporated multiple techniques (e.g. 3D bioprinting, biosensors, artificial intelligence, organoids, online biosensors, gene editing, high-content microscope images, multi-omics, and multi-organs) there might be a boost for OrgOCs chips in multiple applications by creating more complicated next-generation human organoid models simply and efficiently.

Overall, merging state-of-art OrgOCs technology provides a powerful strategy and versatile platform for the biomedical researchers, drug discovery, human disease modeling, and preclinical studies. To promote the biomedical applications of OrgOCs, more synergistic efforts and more cooperation between researchers from different backgrounds are required to help fix the issues mentioned above. We believe that more remarkable, exciting and unprecedented progression will be achieved in OrgOCs for promoting drug screening, personalized medicine, preclinical testing, and systematic studies of pathologies in the future.

## Figures and Tables

**Table 1:** The comparisons of different model systems for biomedicine.

Models	Benefits	Limitations
**2D cell cultures**	• Low equipment dependency and easy to operate for researchers • Minoring basic structure and function, such as tissue barriers/interfaces • Convenient to study mechanisms of drugs or exogenous substances • Easy to conduct high-flux research and large-scale drug screening	• Big differences in genetic and epigenetics information compared to human native tissues. • Fail to recreate the complexity of inter-organ interactions and drug pharmacokinetics • Cannot reflect dynamic physiological conditions and pharmacokinetics of organs in vivo • Difficult for long-term culture to maintain tissue functions
**Animal models**	• Systematically studying the body's response to exogenous substances (e.g., drugs, viruses, vaccines) and pathogenesis • Nonhuman primates are more representative of humans • Some biomedical signals contribute to human studies	• Operation is time-consuming, cumbersome and high-cost • Difficult for* in situ* imaging and on-line monitoring • Difficult to reproduce human biological responses and accurately predict drug safety • No gold standard for extrapolation results from animal models to humans • Limited throughput
**OOCs**	• *In vivo*-like microenvironment elements (e.g., multicellular component, shear stress, mechanical tension, extracellular matrix, fluid) • Representing the human-relevant physiological and pathological features at the organ level • Minoring tissue barriers/interfaces • Studying cell-cell/matrix/virus/drug interaction • Real-time imaging and on-line monitoring • Highly integrating multi-techniques (e.g., biosensors, hydrogels, 3D bioprinting) into organ chips	• Low throughput • Limited cell types can be assembled in a certain space • The hydrophobic PDMS absorbs hydrophobic small molecules, possibly affecting the accuracy and precision of drug detection • No gold standard for commercialization • Low flux for drug screening
**HOs**	• Simulating key features of human native organs or tissues with high fidelity and complex cellular components • Can differentiate into various organoids following given protocols • Long-term preservation of cell phenotype and genotype in vitro • Large-scale omics analysis and drug screening • Greatly contribute to drug discovery and guiding the medications • Available for studies from virus tropism to abundant cell types	• Poorly controlled biochemical and physical environmental signals (e.g., shear stress, mechanical tension, gradient, fluid) • Lack of complicated cellular microenvironment • Relying on ill-defined matrices from animals (e.g. Matrigel) • Low success rate of generating patient-derived organoids • Heterogeneity of tumor organoids derived from patients • Outcomes aren't stable due to individual difference
**OrgOCs**	• Integrating complex microenvironment components (e.g., oxygen gradient, biofactors) with more physiological correlation • High maturation and fidelity of the organoids in structure and function • Real-time monitoring and in situ observation • Alternatives of animal models for drug testing and disease modeling • Cost-effective and time-saving • Systemic responses and long-term toxicity prediction • Expediently realize organoid-organoid communication and organoid-matrix interaction • Accurate prediction of drug response during preclinical stages and phase 1/2 clinical trials • Narrowing the gap between in vitro and in vivo states	• Difficult to successfully construct multi-organoids under the same culture condition • Difficult to long-term coculture of different organoids in an equivalent condition to simulate long-term chronic diseases • Creating maps and standardized evaluation systems in specific disease states is still a problem • Artificial intelligence and computer simulation are needed to identify different forms and phenotypes of organoids in physiological and pathological states • Defined hydrogels are still lacking to replace traditional materials • The hydrophobic PDMS absorbs hydrophobic small molecules, possibly affecting the accuracy and precision of drug detection

**Table 2:** Summary of existing human organoids-on-chips models used for biomedicine.

Types of organoids-on-chips	Organoid sources	Co-cultured cells	ECM	Chip design	Application
**Brain**	• hESCs 145	• N.A.	• Collagen and Laminin	• A two-dimensional compartment consists of a top membrane coupled to a media reservoir and a bottom coverslip	• Modeling the physics of the folding brain
	• hiPSCs 61, 72, 87, 114, 122, 132, 134, 146, 147, 148	• Cerebral organoids, endothelial cells and pericytes 61	• Tissue-derived brain extracellular matrix and Matrigel 146 • Matrigel, Collagenase IV and Gelatin 148 • CaA 134, 147 • Matrigel 61, 72, 87, 114, 132	• A perfusable chip contained parallel channels 72, 87 • Microwell/micropillar array chip 114, 122, 132, 134, 147 • A resin chip with wells, coverslips and channels 61, 148 • A perfusable chip system consists of three layers with five chambers without free-pump 146	• Modeling brain development *in vitro* • Revealing the risk of developing neurodevelopmental disorders due to environmental factors (e.g., nicotine, ethanol, cadmium and prenatal valproic acid) during pregnancy *• In situ* high-resolution time-lapse imaging and cell tracking of brain organoids on-chip
**Lung**	• Lung cancer tissue 136	• N.A.	• Matrigel 136, 149	• A microwell array chip 136, 149	• Assessing drug sensitivity responses
	• Tumor-free tissue 103	• N.A.	• Collagen I	• A sandwiched chip with PET membrane	• Organoid-based expansion of patient-derived primary alveolar type 2 cells for the establishment of preclinical models of the alveolus
**Intestine**	• Duodenum 57	• Macrophages, intestinal myofibroblasts, HUVECs and *C. parvum oocysts*	• Collagen I and Matrigel	• 3D hydrogel-containing microfluidic device	• Modelling tube-shaped mini-intestine and long-term parasite infection
	• Colon 124, 150	• N.A.	• Matrigel	• A microfluidic chip contains a semi-opened microwell array connected by the medium channel and the surrounded pressure channel 124 • A nested array chip consisted of 96 units 150	• Culturing human colon tumor organoids and evaluating nanoparticles under peristalsis • Drug testing in colorectal cancer therapy
	• Colon 137	• Human umbilical vein endothelial cells and primary human lung fibroblasts	• Matrigel, Fibrinogen, Laminin and Collagen IV	• Customizable 384-well IFlowPlate	• Establish vascularized human colon organoid model to explore the cross-talk between the vasculature and the organoids in tissue inflammation
	• Colon 55, 116, 151, 152, 153 • Duodenum 154 • Normal organoid, ulcerative colitis organoid, Crohn's disease organoid, and colorectal cancer organoid 155	• Dendritic cells, T cells, mononuclear phagocytes 116 • HIMECs 151, 153 • HMVEC 152 • Human fecal microbiome 155	• Collagen I and Matrigel 55, 152, 154, 155 • Matrigel, Collagen I and Hydrogel 116 • Collagen IV, Fibronectin and Matrigel 153 • Collagen IV, Matrigel and Fibronectin 151	• A sandwiched PDMS chip with membrane 151, 152, 153, 154, 155 • A GOFlowChip contains two-layer PMMA body, central culture well, silicone gasket and glass coverslip bottom 116	• Investigating the impact of biomechanical and biochemical cues (e.g., morphogen gradient, flow, peristalsis, interleukin 22, Milk Oligosaccharides, niacinamide and tryptophan) on human intestinal morphogenesis and gut barrier function • Molding microbiome or mononuclear phagocyte interactions with the gut epithelium
**Retina**	• hiPSCs 156	• Retinal pigment epithelium	• HA and Matrigel	• A microfluidic chip contains a reservoir and organoid layer, porous membrane layer, and media channel layer	• Analysis of AAV retinal gene therapy vectors Modeling retinopathy induced by anti-malarial and antibiotic drugs
**Spine**	• hESCs 127	• N.A.	• Laminin	• A resin chip consists of a 3D-printed organoid holder and a PC membrane	• Modeling nociceptive circuitry for pain therapeutics discovery
**Breast**	• Endothelialized pancreatic cancer patient 157	• Normal human lung fibroblasts, endothelial cells and immune cells	• Fibrinogen and Matrigel	• A multiplexed platform contains AngioTube scaffolds and a 96-well base plate	• Investigating progression and drug sensitivity in cell lines and patient-derived organoids
**Kidney**	• hiPSCs 77, 158	• Adult human endothelia and fibroblasts 77	• Fibrinogen and Gelatin 77 • Matrigel 158	• A perfusable millifluidic chip using a 3D printer	• Exploring the effect of biochemical and biomechanical factors on vascularization and maturation
**Liver**	• hiPSCs 34, 117, 121	• N.A.	• NaA, Fibrinogen and Cs 117	• A perfusable micropillar chip 34, 121 • All-aqueous droplet microfluidic device 117	• *In situ* generating 3D organoids and modeling nonalcoholic fatty liver disease
**Pancreas/islet**	• hiPSCs 35, 113, 133, 159 • Pancreatic ductal adenocarcinoma cancer patients 160	• N.A.	• NaA and Cs 113 • CaA 133 • Alg/PEI 159 • Matrigel 160	• An all-aqueous microfluidic system 113, 133, 159 • A multilayer microfluidic chip with porous membrane 35 • A reversibly clamped two-layer chamber chip consists of a 200-well array and an overlying layer of fluidic channels 160	• Enabling stem cell organoid engineering to recapitulate organ-specific features in terms structure and function Dynamic and combinatorial drug screening of tumor organoids in an automated way
**Liver-islet axis**	• hiPSCs 10	• N.A.	• N.A.	• A microfluidic device consists of two culture compartments connected by parallel network	• Recapitulating human liver-islet axis for drug therapy of type 2 diabetes
**Liver-heart axis**	• hiPSCs 90, 161	• N.A.	• GelMA, Fibrin and Fibronogen 161	• A chip consists of a top layer, through-hole PDMS layer, PC membrane and bottom layer 90 • Multisensor-integrated microfluidic chip 161	• Assessment of drug responses such as drug metabolism and toxicity
**Liver-stomach- intestine axis**	• Primary hepatocytes, small intestine and corpus stomach 162	• Induced hepatic cells, HUVECs and primary fibroblasts	• Decellularized liver extracellular Matrix	• A microplate array based microffuidic device	• Highthroughput drug screening
**Liver-heart-lung-testis-brain axis**	• Primary cells, hiPSCs and adult testicular tissues 163	• Hepatocytes stellate cells, Kupffer cells, liver endothelial cells, cardiomyocytes, cardiac fibroblast, cardiac endothelium cells, alveolar epithelial A549 cells, lung fibroblasts, bronchial epithelial cells, HUVEC, spermatogonial stem cells, leydig cells, sertoli cells, peritubular cells, brain microvascular endothelial cells (HBMEC), brain vascular pericytes (HBVP), astrocytes, microglial, oligodendrocytes, and neural cells	• Collagen I, human testis ECM, Poly-L-Lysine, Matrigel, HA and Gelatin	• A composite chip consists of PMMA, glass and an adhesive film	• Exploring complex drug and toxin interactions between multi-tissue types in vitro
**Liver-heart-lung-intestine-testis-brain axis**	• Human primary cells or hiPSCs, fresh rabbit colons, adult testicular tissues and normal adult human testicular tissue 164	• Human primary cardiac fibroblasts, lung microvasculature endothelial cells, airway stromal mesenchymal cells, bronchial epithelial cells, HUVECs, rabbit colonic smooth muscle cells, Caco-2 cells, primary spermatogonia, Leydig, sertoli and peritubular myiod cells, brain microvasculature endothelial cells, brain vascular pericytes, astrocytes, microglia, oligodendrocytes, and neurons	• Lung-specific ECM, Collagen I human testis ECM, HA, Gelatin and Fibrin	• A PDMS-free device by layering PMMA and adhesive films with chambers, channels, and ports formed by laser cutting	• Predicting drug efficacy and liver and cardiac toxicity during human Phase I, II and III clinical trials

Human induced pluripotent stem cells (hiPSCs), Human embryonic stem cells (hESCs), Human umbilical vein endothelial cell (HUVEC), Human intestinal microvascular endothelial cells (HIMECs), Human capillary microvascular endothelial cells (HMVEC), Polydimethylsiloxane (PDMS), Poly(methyl methacrylate) (PMMA), HyaluronicAcid (HA), Gelatin methacryloyl (GelMA), Sodium alginate (NaA), Ca-alginate (CaA), Poly(ethylene imine) (PEI), Chitosan (Cs), polycarbonate (PC), polylactic acid (PLA) ,Polyvinyl alcohol (PVA), Dextran sodium sulfate (DSS), Melted poly (ethylene glycol) dimethyl ether (PEGDM), N.A. indicates that the specified feature has not been measured or reported.

## References

[1] Kim SK, Kim YH, Park S, Cho SW (2021). Organoid engineering with microfluidics and biomaterials for liver, lung disease, and cancer modeling. Acta Biomater.

[2] Hoang P, Ma Z (2021). Biomaterial-guided stem cell organoid engineering for modeling development and diseases. Acta Biomater.

[3] Singh AV, Ansari MHD, Rosenkranz D, Maharjan RS, Kriegel FL, Gandhi K (2020). Artificial Intelligence and Machine Learning in Computational Nanotoxicology: Unlocking and Empowering Nanomedicine. Adv Healthc Mater.

[4] Richards B, Tsao D, Zador A (2022). The application of artificial intelligence to biology and neuroscience. Cell.

[5] Pyzer-Knapp EO, Pitera JW, Staar PWJ, Takeda S, Laino T, Sanders DP (2022). Accelerating materials discovery using artificial intelligence, high performance computing and robotics. npj Comput Mater.

[6] Lacombe J, Soldevila M, Zenhausern F (2022). From organ-on-chip to body-on-chip: The next generation of microfluidics platforms for in vitro drug efficacy and toxicity testing. Prog Mol Biol Transl Sci.

[7] Joseph X, Akhil V, Arathi A, Mohanan PV (2022). Comprehensive Development in Organ-On-A-Chip Technology. J Pharm Sci.

[8] Doherty EL, Aw WY, Hickey AJ, Polacheck WJ (2021). Microfluidic and Organ-on-a-Chip Approaches to Investigate Cellular and Microenvironmental Contributions to Cardiovascular Function and Pathology. Front Bioeng Biotechnol.

[9] Cryan JF, O'Riordan KJ, Cowan CSM, Sandhu KV, Bastiaanssen TFS, Boehme M (2019). The microbiota-gut-brain axis. Physiol Rev.

[10] Tao T, Deng P, Wang Y, Zhang X, Guo Y, Chen W (2022). Microengineered multi-organoid system from hiPSCs to recapitulate human liver-islet axis in normal and type 2 diabetes. Adv Sci.

[11] Moysidou CM, Owens RM (2021). Advances in modelling the human microbiome-gut-brain axis in vitro. Biochem Soc Trans.

[12] Hawkins KG, Casolaro C, Brown JA, Edwards DA, Wikswo JP (2020). The Microbiome and the Gut-Liver-Brain Axis for Central Nervous System Clinical Pharmacology: Challenges in Specifying and Integrating In Vitro and In Silico Models. Clin Pharmacol Ther.

[13] Giordano L, Mihaila SM, Eslami Amirabadi H, Masereeuw R (2021). Microphysiological Systems to Recapitulate the Gut-Kidney Axis. Trends Biotechnol.

[14] Li Z, Su W, Zhu Y, Tao T, Li D, Peng X (2017). Drug absorption related nephrotoxicity assessment on an intestine-kidney chip. Biomicrofluidics.

[15] Tenreiro MF, Branco MA, Cotovio JP, Cabral JMS, Fernandes TG, Diogo MM (2023). Advancing organoid design through co-emergence, assembly, and bioengineering. Trends Biotechnol.

[16] Fang G, Chen YC, Lu H, Jin D (2023). Advances in spheroids and organoids on a chip. Adv Funct Mater.

[18] Soldner F, Jaenisch R (2018). Stem Cells, Genome Editing, and the Path to Translational Medicine. Cell.

[19] Tang L (2021). Versatile genome editing. Nat Methods.

[20] van den Brink SC, Alemany A, van Batenburg V, Moris N, Blotenburg M, Vivie J (2020). Single-cell and spatial transcriptomics reveal somitogenesis in gastruloids. Nature.

[21] Fujii M, Matano M, Nanki K, Sato T (2015). Efficient genetic engineering of human intestinal organoids using electroporation. Nat Protoc.

[22] Davenport T, Kalakota R (2019). The potential for artificial intelligence in healthcare. Future Healthc J.

[23] Wong TL, Loh JJ, Lu S, Yan HHN, Siu HC, Xi R (2023). ADAR1-mediated RNA editing of SCD1 drives drug resistance and self-renewal in gastric cancer. Nat Commun.

[24] Wang C, Rabadan Ros R, Martinez-Redondo P, Ma Z, Shi L, Xue Y (2021). In vivo partial reprogramming of myofibers promotes muscle regeneration by remodeling the stem cell niche. Nat Commun.

[25] Kim W, Gwon Y, Park S, Kim H, Kim J (2023). Therapeutic strategies of three-dimensional stem cell spheroids and organoids for tissue repair and regeneration. Bioact Mater.

[26] Wang Y, Wang P, Qin J (2022). Human Organoids and Organs-on-Chips for Addressing COVID-19 Challenges. Adv Sci.

[27] Ren X, Chen W, Yang Q, Li X, Xu L (2022). Patient-derived cancer organoids for drug screening: Basic technology and clinical application. J Gastroenterol Hepatol.

[28] Turhan AG, Hwang JW, Chaker D, Tasteyre A, Latsis T, Griscelli F (2021). iPSC-Derived Organoids as Therapeutic Models in Regenerative Medicine and Oncology. Front Med.

[29] Hofer M, Lutolf MP (2021). Engineering organoids. Nat Rev Mater.

[30] Cui K, Chen T, Zhu Y, Shi Y, Guo Y, Qin J (2023). Engineering placenta-like organoids containing endogenous vascular cells from human-induced pluripotent stem cells. Bioeng Transl Med.

[31] Castiglione H, Vigneron PA, Baquerre C, Yates F, Rontard J, Honegger T (2022). Human Brain Organoids-on-Chip: Advances, Challenges, and Perspectives for Preclinical Applications. Pharm.

[32] Xue Y, Seiler MJ, Tang WC, Wang JY, Delgado J, McLelland BT (2021). Retinal organoids on-a-chip: a micro-millifluidic bioreactor for long-term organoid maintenance. Lab Chip.

[33] Zhang S, Wan Z, Kamm RD (2021). Vascularized organoids on a chip: strategies for engineering organoids with functional vasculature. Lab Chip.

[34] Wang Y, Wang H, Deng P, Tao T, Liu H, Wu S (2020). Modeling Human Nonalcoholic Fatty Liver Disease (NAFLD) with an Organoids-on-a-Chip System. ACS Biomater Sci Eng.

[35] Tao T, Wang Y, Chen W, Li Z, Su W, Guo Y (2019). Engineering human islet organoids from iPSCs using an organ-on-chip platform. Lab Chip.

[36] Huh D, Matthews BD, Mammoto A, Montoya-Zavala M, Hsin HY, Ingber DE (2010). Reconstituting organ-level lung functions on a chip. Science.

[37] Bhatia SN, Ingber DE (2014). Microfluidic organs-on-chips. Nat Biotechnol.

[38] Wang H, Yin F, Li Z, Su W, Li D (2023). Advances of microfluidic lung chips for assessing atmospheric pollutants exposure. Environ Int.

[39] Deng P, Cui K, Shi Y, Zhu Y, Wang Y, Shao X (2022). Fluidic Flow Enhances the Differentiation of Placental Trophoblast-Like 3D Tissue from hiPSCs in a Perfused Macrofluidic Device. Front Bioeng Biotechnol.

[40] Zarrintaj P, Saeb MR, Stadler FJ, Yazdi MK, Nezhad MN, Mohebbi S (2022). Human Organs-on-Chips: A Review of the State-of-the-Art, Current Prospects, and Future Challenges. Adv Biol.

[41] Ronaldson-Bouchard K, Teles D, Yeager K, Tavakol DN, Zhao Y, Chramiec A (2022). A multi-organ chip with matured tissue niches linked by vascular flow. Nat Biomed Eng.

[42] Shuchat S, Yossifon G, Huleihel M (2022). Perfusion in Organ-on-Chip Models and Its Applicability to the Replication of Spermatogenesis In Vitro. Int J Mol Sci.

[43] Chen H, Luo Z, Lin X, Zhu Y, Zhao Y (2023). Sensors-integrated organ-on-a-chip for biomedical applications. Nano Res.

[44] Wang H, Liu H, He F, Chen W, Zhang X, Zhao M (2020). Flexible Generation of Multi-Aqueous Core Hydrogel Capsules Using Microfluidic Aqueous Two-Phase System. Adv Mater Technol.

[45] Zhu Y, Wang H, Yin F, Guo Y, Li F, Gao D (2020). Amnion-on-a-chip: modeling human amniotic development in mid-gestation from pluripotent stem cells. Lab Chip.

[47] Gan Z, Cao Y, Evans RA, Gu M (2013). Three-dimensional deep sub-diffraction optical beam lithography with 9 nm feature size. Nat Commun.

[48] Li J, Wu C, Chu PK, Gelinsky M (2020). 3D printing of hydrogels: Rational design strategies and emerging biomedical applications. Mater Sci Eng R Rep.

[49] Zhou LY, Fu J, He Y (2020). A Review of 3D Printing Technologies for Soft Polymer Materials. Adv Funct Mater.

[50] Quan H, Zhang T, Xu H, Luo S, Nie J, Zhu X (2020). Photo-curing 3D printing technique and its challenges. Bioact Mater.

[51] Zhu Y, Yin F, Wang H, Wang L, Yuan J, Qin J (2018). Placental Barrier-on-a-Chip: Modeling Placental Inflammatory Responses to Bacterial Infection. ACS Biomater Sci Eng.

[52] Shin W, Kim HJ (2018). Intestinal barrier dysfunction orchestrates the onset of inflammatory host-microbiome cross-talk in a human gut inflammation-on-a-chip. Proc Natl Acad Sci U S A.

[53] Yin F, Su W, Wang L, Hu Q (2022). Microfluidic strategies for the blood-brain barrier construction and assessment. Trends Analyt Chem.

[54] Liang D, Su W, Tan M (2022). Advances of microfluidic intestine-on-a-chip for analyzing anti-inflammation of food. Crit Rev Food Sci Nutr.

[55] Shin W, Kim HJ (2022). 3D in vitro morphogenesis of human intestinal epithelium in a gut-on-a-chip or a hybrid chip with a cell culture insert. Nat Protoc.

[56] Zhao M, Liu H, Zhang X, Wang H, Tao T, Qin J (2021). A flexible microfluidic strategy to generate grooved microfibers for guiding cell alignment. Biomater Sci.

[57] Nikolaev M, Mitrofanova O, Broguiere N, Geraldo S, Dutta D, Tabata Y (2020). Homeostatic mini-intestines through scaffold-guided organoid morphogenesis. Nature.

[58] Jing B, Wang ZA, Zhang C, Deng Q, Wei J, Luo Y (2020). Establishment and Application of Peristaltic Human Gut-Vessel Microsystem for Studying Host-Microbial Interaction. Front Bioeng Biotechnol.

[59] Trietsch SJ, Naumovska E, Kurek D, Setyawati MC, Vormann MK, Wilschut KJ (2017). Membrane-free culture and real-time barrier integrity assessment of perfused intestinal epithelium tubes. Nat Commun.

[60] Demers CJ, Soundararajan P, Chennampally P, Cox GA, Briscoe J, Collins SD (2016). Development-on-chip: in vitro neural tube patterning with a microfluidic device. Development.

[61] Salmon I, Grebenyuk S, Abdel Fattah AR, Rustandi G, Pilkington T, Verfaillie C (2022). Engineering neurovascular organoids with 3D printed microfluidic chips. Lab Chip.

[62] Gjorevski N, Nikolaev M, Brown TE, Mitrofanova O, Brandenberg N, DelRio FW (2022). Tissue geometry drives deterministic organoid patterning. Science.

[63] Kim HJ, Li H, Collins JJ, Ingber DE (2016). Contributions of microbiome and mechanical deformation to intestinal bacterial overgrowth and inflammation in a human gut-on-a-chip. Proc Natl Acad Sci U S A.

[64] Lee JS, Romero R, Han YM, Kim HC, Kim CJ, Hong JS (2016). Placenta-on-a-chip: a novel platform to study the biology of the human placenta. J Matern Fetal Neonatal Med.

[65] Yu L, Wei Y, Duan J, Schmitz DA, Sakurai M, Wang L (2021). Blastocyst-like structures generated from human pluripotent stem cells. Nature.

[66] Zheng Y, Xue X, Shao Y, Wang S, Esfahani SN, Li Z (2019). Controlled modelling of human epiblast and amnion development using stem cells. Nature.

[67] Ingber DE (2022). Human organs-on-chips for disease modelling, drug development and personalized medicine. Nat Rev Genet.

[68] Skardal A, Shupe T, Atala A (2016). Organoid-on-a-chip and body-on-a-chip systems for drug screening and disease modeling. Drug Discov Today.

[69] Tuerxun K, He J, Ibrahim I, Yusupu Z, Yasheng A, Xu Q (2022). Bioartificial livers: a review of their design and manufacture. Biofabrication.

[70] Telles-Silva KA, Pacheco L, Komatsu S, Chianca F, Caires-Junior LC, Araujo BHS (2022). Applied Hepatic Bioengineering: Modeling the Human Liver Using Organoid and Liver-on-a-Chip Technologies. Front Bioeng Biotechnol.

[71] Zhu Y, Zhang X, Sun L, Wang Y, Zhao Y (2023). Engineering Human Brain Assembloids by Microfluidics. Adv Mater.

[72] Wang Y, Wang L, Guo Y, Zhu Y, Qin J (2018). Engineering stem cell-derived 3D brain organoids in a perfusable organ-on-a-chip system. RSC Adv.

[73] Zhu Y, Sun L, Wang Y, Cai L, Zhang Z, Shang Y (2022). A Biomimetic Human Lung-on-a-Chip with Colorful Display of Microphysiological Breath. Adv Mater.

[74] Baptista D, Moreira Teixeira L, Barata D, Tahmasebi Birgani Z, King J, van Riet S (2022). 3D Lung-on-Chip Model Based on Biomimetically Microcurved Culture Membranes. ACS Biomater Sci Eng.

[75] Moossavi S, Arrieta MC, Sanati-Nezhad A, Bishehsari F (2022). Gut-on-chip for ecological and causal human gut microbiome research. Trends Microbiol.

[76] Marrero D, Pujol-Vila F, Vera D, Gabriel G, Illa X, Elizalde-Torrent A (2021). Gut-on-a-chip: Mimicking and monitoring the human intestine. Biosens Bioelectron.

[77] Homan KA, Gupta N, Kroll KT, Kolesky DB, Skylar-Scott M, Miyoshi T (2019). Flow-enhanced vascularization and maturation of kidney organoids in vitro. Nat Methods.

[78] Candiello J, Grandhi TSP, Goh SK, Vaidya V, Lemmon-Kishi M, Eliato KR (2018). 3D heterogeneous islet organoid generation from human embryonic stem cells using a novel engineered hydrogel platform. Biomaterials.

[79] Lu RXZ, Lai BFL, Benge T, Wang EY, Davenport Huyer L, Rafatian N (2020). Heart-on-a-Chip Platform for Assessing Toxicity of Air Pollution Related Nanoparticles. Adv Mater Technol.

[80] Occhetta P, Mainardi A, Votta E, Vallmajo-Martin Q, Ehrbar M, Martin I (2019). Hyperphysiological compression of articular cartilage induces an osteoarthritic phenotype in a cartilage-on-a-chip model. Nat Biomed Eng.

[81] Ingber DE (2018). Developmentally inspired human 'organs on chips'. Development.

[82] Zhu Y, Cai L, Chen H, Zhao Y (2022). Developing organs-on-chips for biomedicine. Sci Bull.

[83] Wang Y, Wang P, Qin J (2021). Microfluidic Organs-on-a-Chip for Modeling Human Infectious Diseases. Acc Chem Res.

[84] Osaki T, Sivathanu V, Kamm RD (2018). Vascularized microfluidic organ-chips for drug screening, disease models and tissue engineering. Curr Opin Biotechnol.

[85] Goncalves IM, Carvalho V, Rodrigues RO, Pinho D, Teixeira S, Moita A (2022). Organ-on-a-Chip Platforms for Drug Screening and Delivery in Tumor Cells: A Systematic Review. Cancers.

[86] Kim J, Koo BK, Knoblich JA (2020). Human organoids: model systems for human biology and medicine. Nat Rev Mol Cell Biol.

[87] Wang Y, Wang L, Zhu Y, Qin J (2018). Human brain organoid-on-a-chip to model prenatal nicotine exposure. Lab Chip.

[88] Matsui TK, Tsuru Y, Hasegawa K, Kuwako KI (2021). Vascularization of human brain organoids. Stem Cells.

[89] Zhao D, Lei W, Hu S (2021). Cardiac organoid - a promising perspective of preclinical model. Stem Cell Res Ther.

[90] Yin F, Zhang X, Wang L, Wang Y, Zhu Y, Li Z (2021). HiPSC-derived multi-organoids-on-chip system for safety assessment of antidepressant drugs. Lab Chip.

[91] Sato T, Stange DE, Ferrante M, Vries RG, Van Es JH, Van den Brink S (2011). Long-term expansion of epithelial organoids from human colon, adenoma, adenocarcinoma, and Barrett's epithelium. Gastroenterol.

[92] Rahmani S, Breyner NM, Su HM, Verdu EF, Didar TF (2019). Intestinal organoids: A new paradigm for engineering intestinal epithelium in vitro. Biomaterials.

[93] Eisenstein M (2018). Organoids: the body builders. Nat Methods.

[94] Kim E, Choi S, Kang B, Kong J, Kim Y, Yoon WH (2020). Creation of bladder assembloids mimicking tissue regeneration and cancer. Nature.

[95] Minoli M, Cantore T, Hanhart D, Kiener M, Fedrizzi T, La Manna F (2023). Bladder cancer organoids as a functional system to model different disease stages and therapy response. Nat Commun.

[96] Kim J, Minna JD (2023). Moving toward precision medicine with lung cancer organoids. Cell Rep Med.

[97] Hendriks D, Brouwers JF, Hamer K, Geurts MH, Luciana L, Massalini S (2023). Engineered human hepatocyte organoids enable CRISPR-based target discovery and drug screening for steatosis. Nat Biotechnol.

[98] Velasco S, Kedaigle AJ, Simmons SK, Nash A, Rocha M, Quadrato G (2019). Individual brain organoids reproducibly form cell diversity of the human cerebral cortex. Nature.

[99] Wilson MN, Thunemann M, Liu X, Lu Y, Puppo F, Adams JW (2022). Multimodal monitoring of human cortical organoids implanted in mice reveal functional connection with visual cortex. Nat Commun.

[100] Zou T, Gao L, Zeng Y, Li Q, Li Y, Chen S (2019). Organoid-derived C-Kit(+)/SSEA4(-) human retinal progenitor cells promote a protective retinal microenvironment during transplantation in rodents. Nat Commun.

[101] Puschhof J, Pleguezuelos-Manzano C, Martinez-Silgado A, Akkerman N, Saftien A, Boot C (2021). Intestinal organoid cocultures with microbes. Nat Protoc.

[102] Garcia-Rodriguez I, Sridhar A, Pajkrt D, Wolthers KC (2020). Put Some Guts into It: Intestinal Organoid Models to Study Viral Infection. Viruses.

[103] van Riet S, van Schadewijk A, Khedoe P, Limpens R, Barcena M, Stolk J (2022). Organoid-based expansion of patient-derived primary alveolar type 2 cells for establishment of alveolus epithelial Lung-Chip cultures. Am J Physiol Lung Cell Mol Physiol.

[104] Koning M, Dumas SJ, Avramut MC, Koning RI, Meta E, Lievers E (2022). Vasculogenesis in kidney organoids upon transplantation. npj Regen Med.

[105] Zhu X, Zhang B, He Y, Bao J (2021). Liver Organoids: Formation Strategies and Biomedical Applications. Tissue Eng Regen Med.

[106] Vanslambrouck JM, Wilson SB, Tan KS, Groenewegen E, Rudraraju R, Neil J (2022). Enhanced metanephric specification to functional proximal tubule enables toxicity screening and infectious disease modelling in kidney organoids. Nat Commun.

[107] Liu H, Wang Y, Cui K, Guo Y, Zhang X, Qin J (2019). Advances in Hydrogels in Organoids and Organs-on-a-Chip. Adv Mater.

[108] Neal JT, Li X, Zhu J, Giangarra V, Grzeskowiak CL, Ju J (2018). Organoid Modeling of the Tumor Immune Microenvironment. Cell.

[109] Zhang J, Tavakoli H, Ma L, Li X, Han L, Li X (2022). Immunotherapy discovery on tumor organoid-on-a-chip platforms that recapitulate the tumor microenvironment. Adv Drug Deliv Rev.

[110] Prince E, Cruickshank J, Ba-Alawi W, Hodgson K, Haight J, Tobin C (2022). Biomimetic hydrogel supports initiation and growth of patient-derived breast tumor organoids. Nat Commun.

[111] Chen H, Zhang W, Maskey N, Yang F, Zheng Z, Li C (2022). Urological cancer organoids, patients' avatars for precision medicine: past, present and future. Cell Biosci.

[112] Qu J, Kalyani FS, Liu L, Cheng T, Chen L (2021). Tumor organoids: synergistic applications, current challenges, and future prospects in cancer therapy. Cancer Commun.

[113] Liu H, Wang Y, Wang H, Zhao M, Tao T, Zhang X (2020). A Droplet Microfluidic System to Fabricate Hybrid Capsules Enabling Stem Cell Organoid Engineering. Adv Sci.

[114] Zhu Y, Wang L, Yu H, Yin F, Wang Y, Liu H (2017). In situ generation of human brain organoids on a micropillar array. Lab Chip.

[115] Kim S, Min S, Choi YS, Jo SH, Jung JH, Han K (2022). Tissue extracellular matrix hydrogels as alternatives to Matrigel for culturing gastrointestinal organoids. Nat Commun.

[116] Cherne MD, Sidar B, Sebrell TA, Sanchez HS, Heaton K, Kassama FJ (2021). A Synthetic Hydrogel, VitroGel((R)) ORGANOID-3, Improves Immune Cell-Epithelial Interactions in a Tissue Chip Co-Culture Model of Human Gastric Organoids and Dendritic Cells. Front Pharmacol.

[117] Wang Y, Liu H, Zhang M, Wang H, Chen W, Qin J (2020). One-step synthesis of composite hydrogel capsules to support liver organoid generation from hiPSCs. Biomater Sci.

[118] Rossen NS, Anandakumaran PN, Zur Nieden R, Lo K, Luo W, Park C (2020). Injectable Therapeutic Organoids Using Sacrificial Hydrogels. iScience.

[119] Capeling MM, Czerwinski M, Huang S, Tsai YH, Wu A, Nagy MS (2019). Nonadhesive Alginate Hydrogels Support Growth of Pluripotent Stem Cell-Derived Intestinal Organoids. Stem Cell Rep.

[120] Cruz-Acuna R, Quiros M, Farkas AE, Dedhia PH, Huang S, Siuda D (2017). Synthetic hydrogels for human intestinal organoid generation and colonic wound repair. Nat Cell Biol.

[121] Wang Y, Wang H, Deng P, Chen W, Guo Y, Tao T (2018). In situ differentiation and generation of functional liver organoids from human iPSCs in a 3D perfusable chip system. Lab Chip.

[122] Cui K, Wang Y, Zhu Y, Tao T, Yin F, Guo Y (2020). Neurodevelopmental impairment induced by prenatal valproic acid exposure shown with the human cortical organoid-on-a-chip model. Microsyst Nanoeng.

[123] Cui Y, Xiao R, Zhou Y, Liu J, Wang Y, Yang X (2022). Establishment of organoid models based on a nested array chip for fast and reproducible drug testing in colorectal cancer therapy. Bio-Des Manuf.

[124] Fang G, Lu H, Al-Nakashli R, Chapman R, Zhang Y, Ju LA (2021). Enabling peristalsis of human colon tumor organoids on microfluidic chips. Biofabrication.

[125] Zhao X, Xu Z, Xiao L, Shi T, Xiao H, Wang Y (2021). Review on the Vascularization of Organoids and Organoids-on-a-Chip. Front Bioeng Biotechnol.

[126] Shirure VS, Hughes CCW, George SC (2021). Engineering Vascularized Organoid-on-a-Chip Models. Annu Rev Biomed Eng.

[127] Ao Z, Cai H, Wu Z, Krzesniak J, Tian C, Lai YY (2022). Human Spinal Organoid-on-a-Chip to Model Nociceptive Circuitry for Pain Therapeutics Discovery. Anal Chem.

[128] Yuan R, You D, Wang J, Chen Z, Ge L (2021). A self-healing, antioxidative organoid-chip for cell sorting, capture and release-on-demand. Chem Eng J.

[129] Lim J, Ching H, Yoon JK, Jeon NL, Kim Y (2021). Microvascularized tumor organoids-on-chips: advancing preclinical drug screening with pathophysiological relevance. Nano Converg.

[130] Jantaree P, Bakhchova L, Steinmann U, Naumann M (2021). From 3D Back to 2D Monolayer Stomach Organoids-on-a-Chip. Trends Biotechnol.

[131] Hu Y, Sui X, Song F, Li Y, Li K, Chen Z (2021). Lung cancer organoids analyzed on microwell arrays predict drug responses of patients within a week. Nat Commun.

[132] Yin F, Zhu Y, Wang Y, Qin J (2018). Engineering Brain Organoids to Probe Impaired Neurogenesis Induced by Cadmium. ACS Biomater Sci Eng.

[133] Wang H, Liu H, Zhang X, Wang Y, Zhao M, Chen W (2021). One-Step Generation of Aqueous-Droplet-Filled Hydrogel Fibers as Organoid Carriers Using an All-in-Water Microfluidic System. ACS Appl Mater Interfaces.

[134] Zhu Y, Wang L, Yin F, Yu Y, Wang Y, Liu H (2017). A hollow fiber system for simple generation of human brain organoids. Integr Biol.

[135] Amirifar L, Besanjideh M, Nasiri R, Shamloo A, Nasrollahi F, de Barros NR (2022). Droplet-based microfluidics in biomedical applications. Biofabrication.

[136] Liu Q, Zhao T, Wang X, Chen Z, Hu Y, Chen X (2021). In Situ Vitrification of Lung Cancer Organoids on a Microwell Array. Micromachines.

[137] Rajasekar S, Lin DSY, Abdul L, Liu A, Sotra A, Zhang F (2020). IFlowPlate-A Customized 384-Well Plate for the Culture of Perfusable Vascularized Colon Organoids. Adv Mater.

[138] Sandstrom N, Carannante V, Olofsson K, Sandoz PA, Moussaud-Lamodiere EL, Seashore-Ludlow B (2022). Miniaturized and multiplexed high-content screening of drug and immune sensitivity in a multichambered microwell chip. Cell Rep Methods.

[139] Li ZA, Tuan RS (2022). Towards establishing human body-on-a-chip systems. Stem Cell Res Ther.

[140] Park SE, Georgescu A, Huh D (2019). Organoids-on-a-chip. Science.

[141] Horejs C (2021). Organ chips, organoids and the animal testing conundrum. Nat Rev Mater.

[142] Puschhof J, Pleguezuelos-Manzano C, Clevers H (2021). Organoids and organs-on-chips: Insights into human gut-microbe interactions. Cell Host Microbe.

[143] Gong J, Li M, Kang J, Yin Z, Cha Z, Yang J (2022). Microfluidic Techniques for Next-Generation Organoid Systems. Adv Mater Interfaces.

[144] Mou X, Zhang A, He T, Chen R, Zhou F, Yeung TC (2023). Organoid models for Chinese herbal medicine studies. Acta Materia Medica.

[145] Karzbrun E, Kshirsagar A, Cohen SR, Hanna JH, Reiner O (2018). Human Brain Organoids on a Chip Reveal the Physics of Folding. Nat Phys.

[146] Cho AN, Jin Y, An Y, Kim J, Choi YS, Lee JS (2021). Microfluidic device with brain extracellular matrix promotes structural and functional maturation of human brain organoids. Nat Commun.

[147] Zhu Y, Wang L, Yin F, Yu Y, Wang Y, Shepard MJ (2017). Probing impaired neurogenesis in human brain organoids exposed to alcohol. Integr Biol.

[148] Khan I, Prabhakar A, Delepine C, Tsang H, Pham V, Sur M (2021). A low-cost 3D printed microfluidic bioreactor and imaging chamber for live-organoid imaging. Biomicrofluidics.

[149] Jung DJ, Shin TH, Kim M, Sung CO, Jang SJ, Jeong GS (2019). A one-stop microfluidic-based lung cancer organoid culture platform for testing drug sensitivity. Lab Chip.

[150] Pinho D, Santos D, Vila A, Carvalho S (2021). Establishment of Colorectal Cancer Organoids in Microfluidic-Based System. Micromachines.

[151] Shin W, Ambrosini YM, Shin YC, Wu A, Min S, Koh D (2020). Robust Formation of an Epithelial Layer of Human Intestinal Organoids in a Polydimethylsiloxane-Based Gut-on-a-Chip Microdevice. Front Med Technol.

[152] Shin W, Hinojosa CD, Ingber DE, Kim HJ (2019). Human Intestinal Morphogenesis Controlled by Transepithelial Morphogen Gradient and Flow-Dependent Physical Cues in a Microengineered Gut-on-a-Chip. iScience.

[153] Suligoj T, Vigsnaes LK, Abbeele PVD, Apostolou A, Karalis K, Savva GM (2020). Effects of Human Milk Oligosaccharides on the Adult Gut Microbiota and Barrier Function. Nutrients.

[154] Bein A, Fadel CW, Swenor B, Cao W, Powers RK, Camacho DM (2022). Nutritional deficiency in an intestine-on-a-chip recapitulates injury hallmarks associated with environmental enteric dysfunction. Nat Biomed Eng.

[155] Shin YC, Shin W, Koh D, Wu A, Ambrosini YM, Min S (2020). Three-Dimensional Regeneration of Patient-Derived Intestinal Organoid Epithelium in a Physiodynamic Mucosal Interface-on-a-Chip. Micromachines.

[156] Achberger K, Cipriano M, Duchs MJ, Schon C, Michelfelder S, Stierstorfer B (2021). Human stem cell-based retina on chip as new translational model for validation of AAV retinal gene therapy vectors. Stem Cell Reports.

[157] Shirure VS, Bi Y, Curtis MB, Lezia A, Goedegebuure MM, Goedegebuure SP (2018). Tumor-on-a-chip platform to investigate progression and drug sensitivity in cell lines and patient-derived organoids. Lab Chip.

[158] Lee HN, Choi YY, Kim JW, Lee YS, Choi JW, Kang T (2021). Effect of biochemical and biomechanical factors on vascularization of kidney organoid-on-a-chip. Nano Converg.

[159] He F, Tao T, Liu H, Wang Y, Cui K, Guo Y (2021). Controllable Fabrication of Composite Core-Shell Capsules at Macro-Scale as Organoids Biocarriers. ACS Appl Bio Mater.

[160] Schuster B, Junkin M, Kashaf SS, Romero-Calvo I, Kirby K, Matthews J (2020). Automated microfluidic platform for dynamic and combinatorial drug screening of tumor organoids. Nat Commun.

[161] Zhang YS, Aleman J, Shin SR, Kilic T, Kim D, Mousavi Shaegh SA (2017). Multisensor-integrated organs-on-chips platform for automated and continual in situ monitoring of organoid behaviors. Proc Natl Acad Sci U S A.

[162] Jin Y, Kim J, Lee JS, Min S, Kim S, Ahn D-H (2018). Vascularized Liver Organoids Generated Using Induced Hepatic Tissue and Dynamic Liver-Specific Microenvironment as a Drug Testing Platform. Adv Funct Mater.

[163] Rajan SAP, Aleman J, Wan M, Pourhabibi Zarandi N, Nzou G, Murphy S (2020). Probing prodrug metabolism and reciprocal toxicity with an integrated and humanized multi-tissue organ-on-a-chip platform. Acta Biomater.

[164] Skardal A, Aleman J, Forsythe S, Rajan S, Murphy S, Devarasetty M (2020). Drug compound screening in single and integrated multi-organoid body-on-a-chip systems. Biofabrication.

[165] Yu F, Hunziker W, Choudhury D (2019). Engineering Microfluidic Organoid-on-a-Chip Platforms. Micromachines.

[166] Apostolou A, Panchakshari RA, Banerjee A, Manatakis DV, Paraskevopoulou MD, Luc R (2021). A Novel Microphysiological Colon Platform to Decipher Mechanisms Driving Human Intestinal Permeability. Cell Mol Gastroenterol Hepatol.

[167] Takahashi Y, Noguchi M, Inoue Y, Sato S, Shimizu M, Kojima H (2022). Organoid-derived intestinal epithelial cells are a suitable model for preclinical toxicology and pharmacokinetic studies. iScience.

[168] Lamers MM, van der Vaart J, Knoops K, Riesebosch S, Breugem TI, Mykytyn AZ (2021). An organoid-derived bronchioalveolar model for SARS-CoV-2 infection of human alveolar type II-like cells. EMBO J.

[169] Wang H, Liu H, Liu H, Su W, Chen W, Qin J (2019). One-Step Generation of Core-Shell Gelatin Methacrylate (GelMA) Microgels Using a Droplet Microfluidic System. Adv Mater Technol.

[170] Ouchi R, Togo S, Kimura M, Shinozawa T, Koido M, Koike H (2019). Modeling Steatohepatitis in Humans with Pluripotent Stem Cell-Derived Organoids. Cell Metab.

[171] Sorrentino G, Rezakhani S, Yildiz E, Nuciforo S, Heim MH, Lutolf MP (2020). Mechano-modulatory synthetic niches for liver organoid derivation. Nat Commun.

[172] Ranga A, Girgin M, Meinhardt A, Eberle D, Caiazzo M, Tanaka EM (2016). Neural tube morphogenesis in synthetic 3D microenvironments. Proc Natl Acad Sci U S A.

[173] Sternberg AK, Buck VU, Classen-Linke I, Leube RE (2021). How Mechanical Forces Change the Human Endometrium during the Menstrual Cycle in Preparation for Embryo Implantation. Cells.

[174] Gjorevski N, Sachs N, Manfrin A, Giger S, Bragina ME, Ordonez-Moran P (2016). Designer matrices for intestinal stem cell and organoid culture. Nature.

[175] D'Urso M, Kurniawan NA (2020). Mechanical and Physical Regulation of Fibroblast-Myofibroblast Transition: From Cellular Mechanoresponse to Tissue Pathology. Front Bioeng Biotechnol.

[176] White ES (2015). Lung extracellular matrix and fibroblast function. Ann Am Thorac Soc.

[177] Matsuzaki S, Pouly JL, Canis M (2017). Effects of U0126 and MK2206 on cell growth and re-growth of endometriotic stromal cells grown on substrates of varying stiffness. Sci Rep.

[178] Zhang T, Guo S, Zhou H, Wu Z, Liu J, Qiu C (2021). Endometrial extracellular matrix rigidity and IFNtau ensure the establishment of early pregnancy through activation of YAP. Cell Prolif.

[179] Matsuzaki S (2021). Mechanobiology of the female reproductive system. Reprod Med Biol.

[180] Walther BK, Rajeeva Pandian NK, Gold KA, Kilic ES, Sama V, Gu J (2021). Mechanotransduction-on-chip: vessel-chip model of endothelial YAP mechanobiology reveals matrix stiffness impedes shear response. Lab Chip.

[181] Hushka EA, Yavitt FM, Brown TE, Dempsey PJ, Anseth KS (2020). Relaxation of Extracellular Matrix Forces Directs Crypt Formation and Architecture in Intestinal Organoids. Adv Healthc Mater.

[182] Lewis-Israeli YR, Wasserman AH, Gabalski MA, Volmert BD, Ming Y, Ball KA (2021). Self-assembling human heart organoids for the modeling of cardiac development and congenital heart disease. Nat Commun.

[183] Tan SY, Jing Q, Leung Z, Xu Y, Cheng LKW, Tam SST (2022). Transcriptomic analysis of 3D vasculature-on-a-chip reveals paracrine factors affecting vasculature growth and maturation. Lab Chip.

[184] Huang D, Zhao C, Wen B, Fu X, Shang L, Kong W (2022). Oxygen-carrying microfluidic microcapsules for enhancing chemo-sonodynamic therapy on patient-derived tumor organoid models. Chem Eng J.

[185] Kim R, Attayek PJ, Wang Y, Furtado KL, Tamayo R, Sims CE (2019). An in vitro intestinal platform with a self-sustaining oxygen gradient to study the human gut/microbiome interface. Biofabrication.

[186] Grassart A, Malarde V, Gobaa S, Sartori-Rupp A, Kerns J, Karalis K (2019). Bioengineered Human Organ-on-Chip Reveals Intestinal Microenvironment and Mechanical Forces Impacting Shigella Infection. Cell Host Microbe.

[187] Kavand H, Nasiri R, Herland A (2022). Advanced Materials and Sensors for Microphysiological Systems: Focus on Electronic and Electrooptical Interfaces. Adv Mater.

[188] Buchanan CF, Verbridge SS, Vlachos PP, Rylander MN (2014). Flow shear stress regulates endothelial barrier function and expression of angiogenic factors in a 3D microfluidic tumor vascular model. Cell Adh Migr.

[189] Lee A, Hudson AR, Shiwarski DJ, Tashman JW, Hinton TJ, Yerneni S (2019). 3D bioprinting of collagen to rebuild components of the human heart. Science.

[190] Henry OYF, Villenave R, Cronce MJ, Leineweber WD, Benz MA, Ingber DE (2017). Organs-on-chips with integrated electrodes for trans-epithelial electrical resistance (TEER) measurements of human epithelial barrier function. Lab Chip.

[191] Takahashi K, Tanabe K, Ohnuki M, Narita M, Ichisaka T, Tomoda K (2007). Induction of pluripotent stem cells from adult human fibroblasts by defined factors. Cell.

[192] Cederquist GY, Asciolla JJ, Tchieu J, Walsh RM, Cornacchia D, Resh MD (2019). Specification of positional identity in forebrain organoids. Nat Biotechnol.

[193] Rifes P, Isaksson M, Rathore GS, Aldrin-Kirk P, Møller OK, Barzaghi G (2020). Modeling neural tube development by differentiation of human embryonic stem cells in a microfluidic WNT gradient. Nat Biotechnol.

[194] West RC, Ming H, Logsdon DM, Sun J, Rajput SK, Kile RA (2019). Dynamics of trophoblast differentiation in peri-implantation-stage human embryos. Proc Natl Acad Sci U S A.

[195] Robins JC, Heizer A, Hardiman A, Hubert M, Handwerger S (2007). Oxygen tension directs the differentiation pathway of human cytotrophoblast cells. Placenta.

[196] Quadrato G, Nguyen T, Macosko EZ, Sherwood JL, Yang SM, Berger DR (2017). Cell diversity and network dynamics in photosensitive human brain organoids. Nature.

[197] Martin NR, Passey SL, Player DJ, Mudera V, Baar K, Greensmith L (2015). Neuromuscular Junction Formation in Tissue-Engineered Skeletal Muscle Augments Contractile Function and Improves Cytoskeletal Organization. Tissue Eng Part A.

[198] Vila OF, Uzel SGM, Ma SP, Williams D, Pak J, Kamm RD (2019). Quantification of human neuromuscular function through optogenetics. Theranostics.

[199] Santhanam N, Kumanchik L, Guo X, Sommerhage F, Cai1 Y, Jackson1 M (2018). Stem cell derived phenotypic human neuromuscular junction model for dose response evaluation of therapeutics. Biomaterials.

[200] Tan SY, Leung Z, Wu AR (2020). Recreating Physiological Environments In Vitro: Design Rules for Microfluidic-Based Vascularized Tissue Constructs. Small.

[201] Agarwal P, Wang H, Sun M, Xu J, Zhao S, Liu Z (2017). Microfluidics Enabled Bottom-Up Engineering of 3D Vascularized Tumor for Drug Discovery. ACS Nano.

[202] Xie R, Zheng W, Guan L, Ai Y, Liang Q (2020). Engineering of Hydrogel Materials with Perfusable Microchannels for Building Vascularized Tissues. Small.

[203] Mandrycky CJ, Howard CC, Rayner SG, Shin YJ, Zheng Y (2021). Organ-on-a-chip systems for vascular biology. J Mol Cell Cardiol.

[204] Zhu Y, Sun L, Fu X, Liu J, Liang Z, Tan H (2021). Engineering microcapsules to construct vascularized human brain organoids. Chem Eng J.

[205] Jung O, Tung YT, Sim E, Chen YC, Lee E, Ferrer M (2022). Development of human-derived, three-dimensional respiratory epithelial tissue constructs with perfusable microvasculature on a high-throughput microfluidics screening platform. Biofabrication.

[206] Kim D, Hwang KS, Seo EU, Seo S, Lee BC, Choi N (2022). Vascularized Lung Cancer Model for Evaluating the Promoted Transport of Anticancer Drugs and Immune Cells in an Engineered Tumor Microenvironment. Adv Healthc Mater.

[207] Park D, Lee J, Chung JJ, Jung Y, Kim SH (2020). Integrating Organs-on-Chips: Multiplexing, Scaling, Vascularization, and Innervation. Trends Biotechnol.

[208] Wang X, Sun Q, Pei J (2018). Microfluidic-Based 3D Engineered Microvascular Networks and Their Applications in Vascularized Microtumor Models. Micromachines.

[209] Zhang J, Huang YJ, Yoon JY, Kemmitt J, Wright C, Schneider K (2021). Primary human colonic mucosal barrier crosstalk with super oxygen-sensitive Faecalibacterium prausnitzii in continuous culture. Med.

[210] Humayun M, Chow CW, Young EWK (2018). Microfluidic lung airway-on-a-chip with arrayable suspended gels for studying epithelial and smooth muscle cell interactions. Lab Chip.

[211] Wimmer RA, Leopoldi A, Aichinger M, Wick N, Hantusch B, Novatchkova M (2019). Human blood vessel organoids as a model of diabetic vasculopathy. Nature.

[212] Lebedenko CG, Banerjee IA (2021). Enhancing Kidney Vasculature in Tissue Engineering-Current Trends and Approaches: A Review. Biomimetics.

[213] Lai BFL, Lu RXZ, Davenport Huyer L, Kakinoki S, Yazbeck J, Wang EY (2021). A well plate-based multiplexed platform for incorporation of organoids into an organ-on-a-chip system with a perfusable vasculature. Nat Protoc.

[214] Zhang B, Montgomery M, Chamberlain MD, Ogawa S, Korolj A, Pahnke A (2016). Biodegradable scaffold with built-in vasculature for organ-on-a-chip engineering and direct surgical anastomosis. Nat Mater.

[215] Takehara H, Sakaguchi K, Sekine H, Okano T, Shimizu T (2019). Microfluidic vascular-bed devices for vascularized 3D tissue engineering: tissue engineering on a chip. Biomed Microdevices.

[216] Su H, Li Q, Li D, Li H, Feng Q, Cao X (2022). A versatile strategy to construct free-standing multi-furcated vessels and a complicated vascular network in heterogeneous porous scaffolds via combination of 3D printing and stimuli-responsive hydrogels. Mater Horiz.

[217] Mallott EK, Amato KR (2021). Host specificity of the gut microbiome. Nat Rev Microbiol.

[218] Wastyk HC, Fragiadakis GK, Perelman D, Dahan D, Merrill BD, Yu FB (2021). Gut-microbiota-targeted diets modulate human immune status. Cell.

[219] Jalili-Firoozinezhad S, Gazzaniga FS, Calamari EL, Camacho DM, Fadel CW, Bein A (2019). A complex human gut microbiome cultured in an anaerobic intestine-on-a-chip. Nat Biomed Eng.

[220] Kim HJ, Lee J, Choi JH, Bahinski A, Ingber DE (2016). Co-culture of Living Microbiome with Microengineered Human Intestinal Villi in a Gut-on-a-Chip Microfluidic Device. J Vis Exp.

[221] Ambrosini YM, Shin W, Min S, Kim HJ (2020). Microphysiological Engineering of Immune Responses in Intestinal Inflammation. Immune Netw.

[222] Peterson LW, Artis D (2014). Intestinal epithelial cells: regulators of barrier function and immune homeostasis. Nat Rev Immunol.

[223] Yissachar N, Zhou Y, Ung L, Lai NY, Mohan JF, Ehrlicher A (2017). An Intestinal Organ Culture System Uncovers a Role for the Nervous System in Microbe-Immune Crosstalk. Cell.

[224] Barkal LJ, Procknow CL, Alvarez-Garcia YR, Niu M, Jimenez-Torres JA, Brockman-Schneider RA (2017). Microbial volatile communication in human organotypic lung models. Nat Commun.

[225] Ahlawat S, Asha, Sharma KK (2021). Gut-organ axis: a microbial outreach and networking. Lett Appl Microbiol.

[226] Raimondi MT, Albani D, Giordano C (2019). An Organ-On-A-Chip Engineered Platform to Study the Microbiota-Gut-Brain Axis in Neurodegeneration. Trends Mol Med.

[227] Raimondi I, Izzo L, Tunesi M, Comar M, Albani D, Giordano C (2019). Organ-On-A-Chip in vitro Models of the Brain and the Blood-Brain Barrier and Their Value to Study the Microbiota-Gut-Brain Axis in Neurodegeneration. Front Bioeng Biotechnol.

[228] Wehedy E, Shatat IF, Al Khodor S (2021). The Human Microbiome in Chronic Kidney Disease: A Double-Edged Sword. Front Med.

[229] Trapecar M, Wogram E, Svoboda D, Communal C, Omer A, Lungjangwa T (2021). Human physiomimetic model integrating microphysiological systems of the gut, liver, and brain for studies of neurodegenerative diseases. Sci Adv.

[230] Qian X, Nguyen HN, Song MM, Hadiono C, Ogden SC, Hammack C (2016). Brain-Region-Specific Organoids Using Mini-bioreactors for Modeling ZIKV Exposure. Cell.

[231] Yin F, Zhu Y, Wang H, Wang Y, Li D, Qin J (2020). Microengineered hiPSC-Derived 3D Amnion Tissue Model to Probe Amniotic Inflammatory Responses under Bacterial Exposure. ACS Biomater Sci Eng.

[232] Roodsant T, Navis M, Aknouch I, Renes IB, van Elburg RM, Pajkrt D (2020). A Human 2D Primary Organoid-Derived Epithelial Monolayer Model to Study Host-Pathogen Interaction in the Small Intestine. Front Cell Infect Microbiol.

[233] White CR, Alton LA, Bywater CL, Lombardi EJ, Marshall DJ (2022). Metabolic scaling is the product of life-history optimization. Science.

[234] Guzzardi MA, Vozzi F, Ahluwalia AD (2009). Study of the crosstalk between hepatocytes and endothelial cells using a novel multicompartmental bioreactor: a comparison between connected cultures and cocultures. Tissue Eng Part A.

[235] Wagner I, Materne EM, Brincker S, Sussbier U, Fradrich C, Busek M (2013). A dynamic multi-organ-chip for long-term cultivation and substance testing proven by 3D human liver and skin tissue co-culture. Lab Chip.

[236] Novak R, Ingram M, Marquez S, Das D, Delahanty A, Herland A (2020). Robotic fluidic coupling and interrogation of multiple vascularized organ chips. Nat Biomed Eng.

[237] Jain A, Barrile R, van der Meer AD, Mammoto A, Mammoto T, De Ceunynck K (2018). Primary Human Lung Alveolus-on-a-chip Model of Intravascular Thrombosis for Assessment of Therapeutics. Clin Pharmacol Ther.

[238] Ogden HL, Kim H, Wikenheiser-Brokamp KA, Naren AP, Mun KS (2021). Cystic Fibrosis Human Organs-on-a-Chip. Micromachines.

[239] Ruland L, Andreatta F, Massalini S, Chuva de Sousa Lopes S, Clevers H, Hendriks D (2023). Organoid models of fibrolamellar carcinoma mutations reveal hepatocyte transdifferentiation through cooperative BAP1 and PRKAR2A loss. Nat Commun.

[240] Chen S, Chen X, Geng Z, Su J (2022). The horizon of bone organoid: A perspective on construction and application. Bioact Mater.

[241] Shik Mun K, Arora K, Huang Y, Yang F, Yarlagadda S, Ramananda Y (2019). Patient-derived pancreas-on-a-chip to model cystic fibrosis-related disorders. Nat Commun.

[242] Driehuis E, Kretzschmar K, Clevers H (2020). Establishment of patient-derived cancer organoids for drug-screening applications. Nat Protoc.

[243] Sachs N, de Ligt J, Kopper O, Gogola E, Bounova G, Weeber F (2018). A Living Biobank of Breast Cancer Organoids Captures Disease Heterogeneity. Cell.

[244] Kim M, Mun H, Sung CO, Cho EJ, Jeon HJ, Chun SM (2019). Patient-derived lung cancer organoids as in vitro cancer models for therapeutic screening. Nat Commun.

[245] Kelley KW, Pasca SP (2022). Human brain organogenesis: Toward a cellular understanding of development and disease. Cell.

[246] Caiazza C, Parisi S, Caiazzo M (2021). Liver Organoids: Updates on Disease Modeling and Biomedical Applications. Biol.

[247] Boretto M, Maenhoudt N, Luo X, Hennes A, Boeckx B, Bui B (2019). Patient-derived organoids from endometrial disease capture clinical heterogeneity and are amenable to drug screening. Nat Cell Biol.

[248] Yao Y, Xu X, Yang L, Zhu J, Wan J, Shen L (2020). Patient-Derived Organoids Predict Chemoradiation Responses of Locally Advanced Rectal Cancer. Cell Stem Cell.

[249] Gronholm M, Feodoroff M, Antignani G, Martins B, Hamdan F, Cerullo V (2021). Patient-Derived Organoids for Precision Cancer Immunotherapy. Cancer Res.

[250] Liu L, Yu L, Li Z, Li W, Huang W (2021). Patient-derived organoid (PDO) platforms to facilitate clinical decision making. J Transl Med.

[251] Bae J, Choi YS, Cho G, Jang SJ (2022). The Patient-Derived Cancer Organoids: Promises and Challenges as Platforms for Cancer Discovery. Cancers.

[252] Nilsson Hall G, Mendes LF, Gklava C, Geris L, Luyten FP, Papantoniou I (2020). Developmentally Engineered Callus Organoid Bioassemblies Exhibit Predictive In Vivo Long Bone Healing. Adv Sci.

[253] Kasendra M, Tovaglieri A, Sontheimer-Phelps A, Jalili-Firoozinezhad S, Bein A, Chalkiadaki A (2018). Development of a primary human Small Intestine-on-a-Chip using biopsy-derived organoids. Sci Rep.

[254] Wang L, Tao T, Su W, Yu H, Yu Y, Qin J (2017). A disease model of diabetic nephropathy in a glomerulus-on-a-chip microdevice. Lab Chip.

[255] Kukla DA, Khetani SR (2021). Bioengineered Liver Models for Investigating Disease Pathogenesis and Regenerative Medicine. Semin Liver Dis.

[256] Benam KH, Novak R, Nawroth J, Hirano-Kobayashi M, Ferrante TC, Choe Y (2016). Matched-Comparative Modeling of Normal and Diseased Human Airway Responses Using a Microengineered Breathing Lung Chip. Cell Syst.

[257] McDuffie D, Barr D, Helm M, Baumert T, Agarwal A, Thomas E (2023). Physiomimetic In Vitro Human Models for Viral Infection in the Liver. Semin Liver Dis.

[258] Bost P, Giladi A, Liu Y, Bendjelal Y, Xu G, David E (2020). Host-Viral Infection Maps Reveal Signatures of Severe COVID-19 Patients. Cell.

[259] Zhang X, Chen Q, Xu G (2023). Clinical manifestations of COVID-19 infection in dialysis patients and protective effect of COVID-19 vaccine. Inflamm Res.

[260] Shpichka A, Bikmulina P, Peshkova M, Kosheleva N, Zurina I, Zahmatkesh E (2020). Engineering a Model to Study Viral Infections: Bioprinting, Microfluidics, and Organoids to Defeat Coronavirus Disease 2019 (COVID-19). Int J Bioprint.

[261] Natarajan V, Simoneau CR, Erickson AL, Meyers NL, Baron JL, Cooper S (2022). Modelling T-cell immunity against hepatitis C virus with liver organoids in a microfluidic coculture system. Open Biol.

[262] Saygili E, Yildiz-Ozturk E, Green MJ, Ghaemmaghami AM, Yesil-Celiktas O (2021). Human lung-on-chips: Advanced systems for respiratory virus models and assessment of immune response. Biomicrofluidics.

[263] Zhang M, Wang P, Luo R, Wang Y, Li Z, Guo Y (2020). Biomimetic Human Disease Model of SARS-CoV-2 Induced Lung Injury and Immune Responses on Organ Chip System. Adv Sci.

[264] Villenave R, Wales SQ, Hamkins-Indik T, Papafragkou E, Weaver JC, Ferrante TC (2017). Human Gut-On-A-Chip Supports Polarized Infection of Coxsackie B1 Virus In Vitro. PLoS One.

[265] Sachs N, Papaspyropoulos A, Zomer-van Ommen DD, Heo I, Bottinger L, Klay D (2019). Long-term expanding human airway organoids for disease modeling. EMBO J.

[266] Upadhya S, Rehman J, Malik AB, Chen S (2022). Mechanisms of Lung Injury Induced by SARS-CoV-2 Infection. Physiol.

[267] Robinot R, Hubert M, de Melo GD, Lazarini F, Bruel T, Smith N (2021). SARS-CoV-2 infection induces the dedifferentiation of multiciliated cells and impairs mucociliary clearance. Nat Commun.

[268] Grau-Exposito J, Sanchez-Gaona N, Massana N, Suppi M, Astorga-Gamaza A, Perea D (2021). Peripheral and lung resident memory T cell responses against SARS-CoV-2. Nat Commun.

[269] Hysenaj L, Little S, Kulhanek K, Gbenedio OM, Rodriguez L, Shen A SARS-CoV-2 infection studies in lung organoids identify TSPAN8 as novel mediator. bioRxiv. 2021: 446640.

[270] Li F, Han M, Dai P, Xu W, He J, Tao X (2021). Distinct mechanisms for TMPRSS2 expression explain organ-specific inhibition of SARS-CoV-2 infection by enzalutamide. Nat Commun.

[271] Salahudeen AA, Choi SS, Rustagi A, Zhu J, van Unen V, de la OS (2020). Progenitor identification and SARS-CoV-2 infection in human distal lung organoids. Nature.

[272] Guo Y, Luo R, Wang Y, Deng P, Song T, Zhang M (2021). SARS-CoV-2 induced intestinal responses with a biomimetic human gut-on-chip. Sci Bull.

[273] Ridings JE (2013). The thalidomide disaster, lessons from the past. Methods Mol Biol.

[274] Loiodice S, Nogueira da Costa A, Atienzar F (2019). Current trends in in silico, in vitro toxicology, and safety biomarkers in early drug development. Drug Chem Toxicol.

[275] Peterson NC, Mahalingaiah PK, Fullerton A, Di Piazza M (2020). Application of microphysiological systems in biopharmaceutical research and development. Lab Chip.

[276] Syama S, Mohanan PV (2021). Microfluidic based human-on-a-chip: A revolutionary technology in scientific research. Trends Food Sci Technol.

[277] Jacob F, Salinas RD, Zhang DY, Nguyen PTT, Schnoll JG, Wong SZH (2020). A Patient-Derived Glioblastoma Organoid Model and Biobank Recapitulates Inter- and Intra-tumoral Heterogeneity. Cell.

[278] Kawasaki K, Toshimitsu K, Matano M, Fujita M, Fujii M, Togasaki K (2020). An Organoid Biobank of Neuroendocrine Neoplasms Enables Genotype-Phenotype Mapping. Cell.

[279] Li Z, Guo Y, Yu Y, Xu C, Xu H, Qin J (2016). Assessment of metabolism-dependent drug efficacy and toxicity on a multilayer organs-on-a-chip. Integr Biol.

[280] Li Z, Jiang L, Tao T, Su W, Guo Y, Yu H (2017). Assessment of cadmium-induced nephrotoxicity using a kidney-on-a-chip device. Toxicol Res.

[281] Jie M, Lin H, He Z, Liu H, Li H, Lin J (2017). An on-chip intestine-liver model for multiple drugs absorption and metabolism behavior simulation. Sci China Chem.

[282] Herland A, Maoz BM, Das D, Somayaji MR, Prantil-Baun R, Novak R (2020). Quantitative prediction of human pharmacokinetic responses to drugs via fluidically coupled vascularized organ chips. Nat Biomed Eng.

[283] Lee BE, Kim DK, Lee H, Yoon S, Park SH, Lee S (2021). Recapitulation of First Pass Metabolism Using 3D Printed Microfluidic Chip and Organoid. Cells.

[284] Ferrari E, Rasponi M (2021). Liver-Heart on chip models for drug safety. APL Bioeng.

[285] Wenzel C, Riefke B, Grundemann S, Krebs A, Christian S, Prinz F (2014). 3D high-content screening for the identification of compounds that target cells in dormant tumor spheroid regions. Exp Cell Res.

[286] Klameth L, Rath B, Hochmaier M, Moser D, Redl M, Mungenast F (2017). Small cell lung cancer: model of circulating tumor cell tumorospheres in chemoresistance. Sci Rep.

[287] Shi X, Li Y, Yuan Q, Tang S, Guo S, Zhang Y (2022). Integrated profiling of human pancreatic cancer organoids reveals chromatin accessibility features associated with drug sensitivity. Nat Commun.

[288] Soto F, Guimaraes CF, Reis RL, Franco W, Rizvi I, Demirci U (2021). Emerging biofabrication approaches for gastrointestinal organoids towards patient specific cancer models. Cancer Lett.

[289] Liu H, Zhang X, Liu J, Qin J (2023). Vascularization of engineered organoids. BMEMat.

[290] Okkelman IA, Neto N, Papkovsky DB, Monaghan MG, Dmitriev RI (2020). A deeper understanding of intestinal organoid metabolism revealed by combining fluorescence lifetime imaging microscopy (FLIM) and extracellular flux analyses. Redox Biol.

